# Platelet transfusions in adult ICU patients with thrombocytopenia: A sub-study of the PLOT-ICU inception cohort study

**DOI:** 10.1111/aas.14467

**Published:** 2024-06-05

**Authors:** Carl Thomas Anthon, Frédéric Pène, Anders Perner, Elie Azoulay, Kathryn Puxty, Andry Van De Louw, Sanjay Chawla, Pedro Castro, Pedro Povoa, Luis Coelho, Victoria Metaxa, Matthias Kochanek, Tobias Liebregts, Thomas Kander, Mirka Sivula, Jo Bønding Andreasen, Lene Bjerregaard Nielsen, Christine Lodberg Hvas, Etienne Dufranc, Emmanuel Canet, Christopher John Wright, Julien Schmidt, Fabrice Uhel, Louai Missri, Mette Krag, Elisabet Cos Badia, Cándido Díaz-Lagares, Sophie Menat, Guillaume Voiriot, Niels Erikstrup Clausen, Kristian Lorentzen, Reidar Kvåle, Andreas Barratt-Due, Thomas Hildebrandt, Aleksander Rygh Holten, Kristian Strand, Morten Heiberg Bestle, Pål Klepstad, Damien Vimpere, Carolina Paulino, Catherina Lueck, Christian Svendsen Juhl, Carolina Costa, Per Martin Bådstøløkken, Lia Susana Aires Lêdo, Morten Hylander Møller, Lene Russell

**Affiliations:** 1 Department of Intensive Care, Copenhagen University Hospital – Rigshospitalet, Copenhagen, Denmark; 2 Médecine Intensive & Réanimation, Hôpital Cochin, Assistance Publique – Hôpitaux de Paris, Institut Cochin, INSERM U1016, CNRS UMR8104, Université Paris Cité, Paris, France; 3 Department of Clinical Medicine, University of Copenhagen, Copenhagen, Denmark; 4 Médecine Intensive & Réanimation, Hôpital Saint-Louis, Assistance Publique – Hôpitaux de Paris, Université Paris Cité, Paris, France; 5 Department of Intensive Care, Glasgow Royal Infirmary, Glasgow, UK; 6 Division of Pulmonary and Critical Care, Penn State University College of Medicine, Hershey, Pennsylvania, USA; 7 Critical Care Medicine Service, Department of Anesthesiology & Critical Care Medicine, Memorial Sloan Kettering Cancer Center, New York, New York, USA; 8 Department of Anesthesiology, Weill Cornell Medical College, New York, New York, USA; 9 Medical Intensive Care Unit, Hospital Clinic of Barcelona; IDIBAPS, University of Barcelona, Barcelona, Spain; 10 Department of Intensive Care, Sao Francisco Xavier Hospital, CHLO, Lisbon, Portugal; 11 Nova Medical School, CHRC, New University of Lisbon, Lisbon, Portugal; 12 Center for Clinical Epidemiology and Research Unit of Clinical Epidemiology, Odense University Hospital, Odense, Denmark; 13 Department of Critical Care, King’s College Hospital NHS Foundation Trust, London, UK; 14 Department I of Internal Medicine, Faculty of Medicine and University Hospital Cologne, University of Cologne, Cologne, Germany; 15 Department of Hematology and Stem Cell Transplantation, University Hospital Essen, University of Duisburg-Essen, Essen, Germany; 16 Department of Intensive and Perioperative Care, Skåne University Hospital, Lund, Sweden; 17 Department of Clinical Sciences, Lund University, Lund, Sweden; 18 Department of Perioperative and Intensive Care Medicine, University of Helsinki and Helsinki University Hospital, Helsinki, Finland; 19 Coagulation Disorders Unit, Department of Hematology, University of Helsinki and Helsinki University Hospital, Helsinki, Finland; 20 Department of Anaesthesia and Intensive Care, Aalborg University Hospital, Aalborg, Denmark; 21 Department of Anaesthesiology and Intensive Care, Odense University Hospital, Odense, Denmark; 22 Department of Anaesthesiology and Intensive Care, Aarhus University Hospital, Aarhus, Denmark; 23 Service de Médecine Intensive Réanimation, Hôpitaux Universitaires Henri-Mondor, Assistance Publique – Hôpitaux de Paris, Paris, France; 24 Médecine Intensive Réanimation, CHU de Nantes, Université de Nantes, Nantes, France; 25 Critical Care Unit, Queen Elizabeth University Hospital, Glasgow, UK; 26 Service de réanimation médico-chirurgicale, Hôpital Avicenne, Assistance Publique – Hôpitaux de Paris, Paris, France; 27 Médecine Intensive Réanimation, Hôpital Louis Mourier, Assistance Publique – Hôpitaux de Paris, DMU ESPRIT, Paris, France; 28 Université Paris Cité, INSERM UMR-S1151, CNRS UMR-S8253, Institut Necker-Enfants Malades, Paris, France; 29 Service de Médecine Intensive-Réanimation, Hôpital Saint-Antoine, Assistance Publique – Hôpitaux de Paris, Sorbonne Université, Paris, France; 30 Department of Intensive Care, Holbaek Hospital, Holbaek, Denmark; 31 Department of Intensive Care, Hospital General Granollers, Barcelona, Spain; 32 Intensive Care Department, Vall d’Hebron Hospital Universitari, Barcelona, Spain; 33 SODIR Research Group, Vall d’Hebron Institut de Recerca (VHIR), Vall d’Hebron Barcelona Hospital Campus, Barcelona, Spain; 34 Service de Médecine Intensive-Réanimation, Hôpital Pitié-Salpêtrière, Assistance Publique – Hôpitaux de Paris, Sorbonne Université, Paris, France; 35 Service de Médecine Intensive Réanimation, Hôpital Tenon, Assistance Publique – Hôpitaux de Paris, Sorbonne Université, Centre de Recherche Saint-Antoine UMRS_938 INSERM, Paris, France; 36 Department of Anaesthesia and Intensive Care, Copenhagen University Hospital – Bispebjerg and Frederiksberg, Copenhagen, Denmark; 37 Department of Intensive Care, Copenhagen University Hospital – Herlev and Gentofte, Herlev, Denmark; 38 Department of Anaesthesia and Intensive Care, Haukeland University Hospital, Bergen, Norway; 39 Faculty of Medicine, University of Bergen, Bergen, Norway; 40 Department of Anaesthesia and Intensive Care Medicine, Division of Emergencies and Critical Care, Rikshospitalet, Oslo University Hospital, Oslo, Norway; 41 Department of Intensive Care, Zealand University Hospital – Roskilde, Roskilde, Denmark; 42 Department of Acute Medicine, Oslo University Hospital, Oslo, Norway; 43 Institute of Clinical Medicine, University of Oslo, Oslo, Norway; 44 Department of Intensive Care, Stavanger University Hospital, Stavanger, Norway; 45 Department of Anaesthesiology and Intensive Care, Copenhagen University Hospital – North Zealand, Hilleroed, Denmark; 46 Department of Intensive Care Medicine, St. Olav’s University Hospital, Trondheim, Norway; 47 Department of Circulation and Medical Imaging, Norwegian University of Technology and Science, Trondheim, Norway; 48 Médecine Intensive & Réanimation, Hôpital Necker, Assistance Publique – Hôpitaux de Paris, Université Paris Cité, Paris, France; 49 Department of Intensive Care, Hospital da Luz Lisboa, Lisbon, Portugal; 50 Department of Hematology, Hemostasis, Oncology and Stem Cell Transplantation, Hannover Medical School, Hannover, Germany; 51 Department of Anaesthesiology, Copenhagen University Hospital – Amager and Hvidovre, Hvidovre, Denmark; 52 Intensive Care Unit, Hospital Professor Doutor Fernando Fonseca, EPE, Amadora, Portugal; 53 Department of Intensive Care B203, Akershus University Hospital, Lorenskog, Norway; 54 Department of Intensive Care Medicine – Unit 2, Hospital Egas Moniz – CHLO, EPE, Lisbon, Portugal

**Keywords:** critical illness, intensive care unit, platelet transfusion, thrombocytopenia

## Abstract

**Background:**

Platelet transfusions are frequently used in the intensive care unit (ICU), but current practices including used product types, volumes, doses and effects are unknown.

**Study design and methods:**

Sub-study of the inception cohort study ‘Thrombocytopenia and Platelet Transfusions in the ICU (PLOT-ICU)’, including acutely admitted, adult ICU patients with thrombocytopenia (platelet count <150 × 10^9^/L). The primary outcome was the number of patients receiving platelet transfusion in ICU by product type. Secondary outcomes included platelet transfusion details, platelet increments, bleeding, other transfusions and mortality.

**Results:**

Amongst 504 patients with thrombocytopenia from 43 hospitals in 10 countries in Europe and the United States, 20.8% received 565 platelet transfusions; 61.0% received pooled products, 21.9% received apheresis products and 17.1% received both with a median of 2 (interquartile range 1–4) days from admission to first transfusion. The median volume per transfusion was 253 mL (180–308 mL) and pooled products accounted for 59.1% of transfusions, however, this varied across countries. Most centres (73.8%) used fixed dosing (medians ranging from 2.0 to 3.5 × 10^11^ platelets/transfusion) whilst some (mainly in France) used weight-based dosing (ranging from 0.5 to 0.7 × 10^11^ platelets per 10 kg body weight). The median platelet count increment for a single prophylactic platelet transfusion was 2 (−1 to 8) × 10^9^/L. Outcomes of patients with thrombocytopenia who did and did not receive platelet transfusions varied.

**Conclusions:**

Among acutely admitted, adult ICU patients with thrombocytopenia, 20.8% received platelet transfusions in ICU of whom most received pooled products, but considerable variation was observed in product type, volumes and doses across countries. Prophylactic platelet transfusions were associated with limited increases in platelet counts.

## 1 | BACKGROUND

Thrombocytopenia (platelet count <150 × 10^9^/L) occurs in approximately 40% of critically ill patients admitted to the intensive care unit (ICU)^1–4^ and may increase the risk of bleeding.^5–7^ Platelet transfusions are widely used to treat or mitigate the risk of bleeding in ICU patients with thrombocytopenia,^3,4,8^ and 5%–24% of all issued platelet transfusions are administered in ICUs.^9,10^

Despite the widespread use of platelet transfusions, there is a paucity of evidence describing current platelet transfusion practice in general ICU patients. While various studies have reported on indications, pre-transfusion platelet counts and platelet increments,^8,11–14^ international data describing the type of platelet product used, platelet transfusion volumes, dosing (i.e. fixed or weight-adjusted) and timing of transfusion remain scarce. The evidence guiding the choice between apheresis-derived and whole-blood-derived platelet transfusion^15–17^ and the appropriate platelet dose^18–20^ is limited and primarily derived from the haematological setting. Together with numerous manufacturing methods^21^ and organisational variation in transfusion services at national, regional or local levels,^22^ considerable practice variation seems likely but remains to be assessed.

We recently conducted a large international cohort study on thrombocytopenia in acutely admitted adult ICU patients.^4^ To inform future platelet transfusion trials, the primary aim of this sub-study was to provide insights into the platelet products, volumes, and doses used, and to map potential differences across and within countries. Secondary aims included describing the characteristics and outcomes of thrombocytopenic ICU patients receiving platelet transfusions. We hypothesised that platelet transfusion practices would vary and that transfused patients would have worse outcomes.

## 2 | METHODS

### 2.1 | Design

This descriptive, sub-study of the PLOT-ICU study was conducted according to a protocol and statistical analysis plan published after the completion of the original study.^23^ Additions to the protocol are available in Data S1. The manuscript was prepared in accordance with the Strengthening the Reporting of Observational Studies in Epidemiology (STROBE) statement as applicable (checklist in Data S1).^24^

### 2.2 | The PLOT-ICU study

The PLOT-ICU study was an international, inception cohort study enrolling 1166 patients from 43 centres across Denmark, Finland, France, Germany, Norway, Portugal, Spain, Sweden, the United Kingdom and the United States. All relevant approvals were obtained before study initiation, and informed consent was collected from patients and/or relatives if required.^4,25^ Participating ICUs conveniently chose a 2-week inception period between May 2021 and July 2022, during which consecutive patients were enrolled at ICU admission and subsequently followed daily during ICU stay for a maximum of 90 days.^4,25^

### 2.3 | Population

The PLOT-ICU study included acutely admitted, adult (≥18 years) ICU patients and excluded patients who had undergone elective open-heart surgery and those who denied informed consent or had already been included.^4,25^ All patients with thrombocytopenia were eligible for this sub-study.^23^

### 2.4 | Definitions

#### 2.4.1 | Thrombocytopenia

Thrombocytopenia and severe thrombocytopenia were defined as patients with a platelet count <150 × 10^9^/L and <50 × 10^9^/L at ICU admission and/or during ICU stay respectively.

#### 2.4.2 | Platelet transfusions

In this study, only platelet transfusions administered in the ICU were considered. Platelet transfusions used in operating rooms were quantified, but not assessed in detail. The number of patients receiving platelet transfusions in operating rooms is described in Data S1.

Platelet transfusions derived from a single donor by plateletpheresis were considered apheresis products, while those derived by pooling platelet concentrates from multiple donors were considered pooled products.

Indications for platelet transfusions were defined as prophylactic (reducing bleeding risk), pre-procedural (covering invasive procedures) and therapeutic (treating bleeding).

Employment of standard platelet doses, administered independent of patient weight, was defined as fixed dosing and dosing adjusted for the patient’s weight was defined as weight-based dosing.

#### 2.4.3 | Bleeding

Major bleeding was defined as a modified World Health Organization (WHO) grade 3 or 4 bleeding.^23^ This included bleeding from critical sites (e.g. central nervous bleeding), fatal bleeding and bleeding requiring either (1) transfusions with red blood cells, (2) intubation and mechanical ventilation or (3) surgical intervention.^23^ Bleedings confined to operating rooms were not assessed in the PLOT-ICU study.^4,25^

### 2.5 | Data

Details on data collection and variables are available elsewhere^4,23,25^ and in Data S1. In brief, baseline data were collected at ICU admission and outcome data were registered daily during ICU stay and on day 90. In addition, we distributed a short survey detailing types of available platelet products, manufacturing methods, dosing practices and average number of platelets per platelet transfusion among all centres participating in the original PLOT-ICU study.^23^

### 2.6 | Outcomes

The primary outcome was the number of patients receiving platelet transfusions in ICU, reported according to the type of product. Secondary platelet transfusion outcomes included types of platelet transfusions used, transfusion volumes, absolute platelet increments for prophylactic platelet transfusions, timing of platelet transfusions and number of centres employing fixed and weight-based dosing. Secondary clinical outcomes included number of patients receiving red blood cell transfusions, number of patients receiving plasma transfusions, number of patients with major bleeding in ICU and thrombosis in ICU and 90-day mortality. Additional details are provided in the protocol^23^ and Data S1.

### 2.7 | Statistical considerations

#### 2.7.1 | Sample size

A sample size calculation was not applicable as the sample size was fixed by design.

#### 2.7.2 | Statistics

All data are presented descriptively; categorical data as numbers and percentages and continuous data as medians and interquartile ranges (IQR). Baseline data and secondary clinical outcomes were stratified according to platelet transfusion status and severe thrombocytopenia.^4^ All analyses were conducted in R version 4.2.0 (R Core Team, R Foundation for Statistical Computing, Vienna, Austria).

##### Assumptions

Platelet transfusion volumes were calculated separately for each product type based on daily totals: that is, the total volume received on a given day divided by the corresponding number of transfusions administered that day. We computed platelet increments for single prophylactic platelet transfusions as the absolute difference between pre-transfusion platelet counts and the lowest platelet count the following day on days with no other intervening platelet transfusions. As the PLOT-ICU study was designed to report on the occurrence of thrombocytopenia, only the lowest platelet count was collected each day. If centres reported platelet doses as means, we assumed normality of the underlying data and interpreted these as medians to facilitate data aggregation.

##### Reporting

The primary outcome, types of platelet transfusions, platelet transfusion volumes and platelet increments are reported as overall results and stratified by countries with ≥10 events. Types of platelet transfusions and platelet transfusion volumes are also reported stratified on sites with ≥10 events in countries with two or more sites with ≥10 events.

We mapped timing and quantity of platelet transfusions in ICU according to the days since ICU admission using heatmaps.

##### Sensitivity analyses

Where possible, we included data from transfusions used in operating rooms (analyses of volumes and timing). In the analysis of platelet increments, we conducted a pre-planned sensitivity analysis excluding transfusions registered in proximity to bleeding or surgery (i.e. any bleeding [WHO grade 1–4] or surgery on the day of transfusion or the next) and a post-hoc analysis excluding transfusions in patients with presumed hypo-proliferative thrombocytopenia defined as patients with haematological malignancy and/or those treated with haematopoietic stem cell transplantation and/or chemotherapy because these patients may be prone to low platelet increments.^26,27^

##### Missing data

We performed modified complete case analysis and reported numbers and percentages of missing data where relevant (details in Data S1).

## 3 | RESULTS

We included 504 patients with thrombocytopenia from the PLOT-ICU study, 134 (26.6%) of whom had severe thrombocytopenia.^4^

Baseline characteristics stratified by platelet transfusion status in ICU and severe thrombocytopenia are presented in Table 1. A high proportion of patients without severe thrombocytopenia who received platelet transfusion were admitted from operating rooms or postoperative facilities with haemorrhage as reason for ICU admission. Patients with severe thrombocytopenia who received platelet transfusions were often admitted from general hospital wards and many had haematological malignancy. Septic shock was common among patients with severe thrombocytopenia irrespective of platelet transfusion status.

### 3.1 | Primary outcome

In total, 105 of 504 patients (20.8%) received platelet transfusion in ICU; 64 (61.0%) of whom received pooled products, 23 (21.9%) received apheresis products and 18 (17.1%) received both products. Proportions of patients receiving pooled, apheresis and both products ranged from 7.1% to 100%, 0.0% to 92.9% and 0.0% to 48% respectively, across countries (eTable 1 in Data S1). The overall median number of platelet transfusions received in ICU was 3 (IQR 1–5); patients transfused with pooled, apheresis and both products received 2 (1–4), 3 (1–4) and 8 (3–14) transfusions, respectively.

### 3.2 | Secondary platelet transfusion outcomes

#### 3.2.1 | Types of platelet transfusions

A total of 565 platelet transfusions were used in ICU; 334 were pooled products (59.1%), and 231 (40.9%) were apheresis products. Proportions of pooled and apheresis products varied between and to some degree within countries (eFigures 1 and 2 in Data S1).

#### 3.2.2 | Platelet transfusion volumes

Overall, the median volume was 253 (IQR 180–308) mL per transfusion. Median volumes varied between countries from 180 to 380 mL (eFigure 3 in Data S1) and to some degree within countries (eFigure 4 in Data S1). Sensitivity analysis including platelet transfusions used in operating rooms produced similar results (eFigures 5 and 6 in Data S1). Volume data were missing for 10/565 (1.8%) ICU platelet transfusions.

#### 3.2.3 | Absolute platelet increments

**FIGURE 1:**
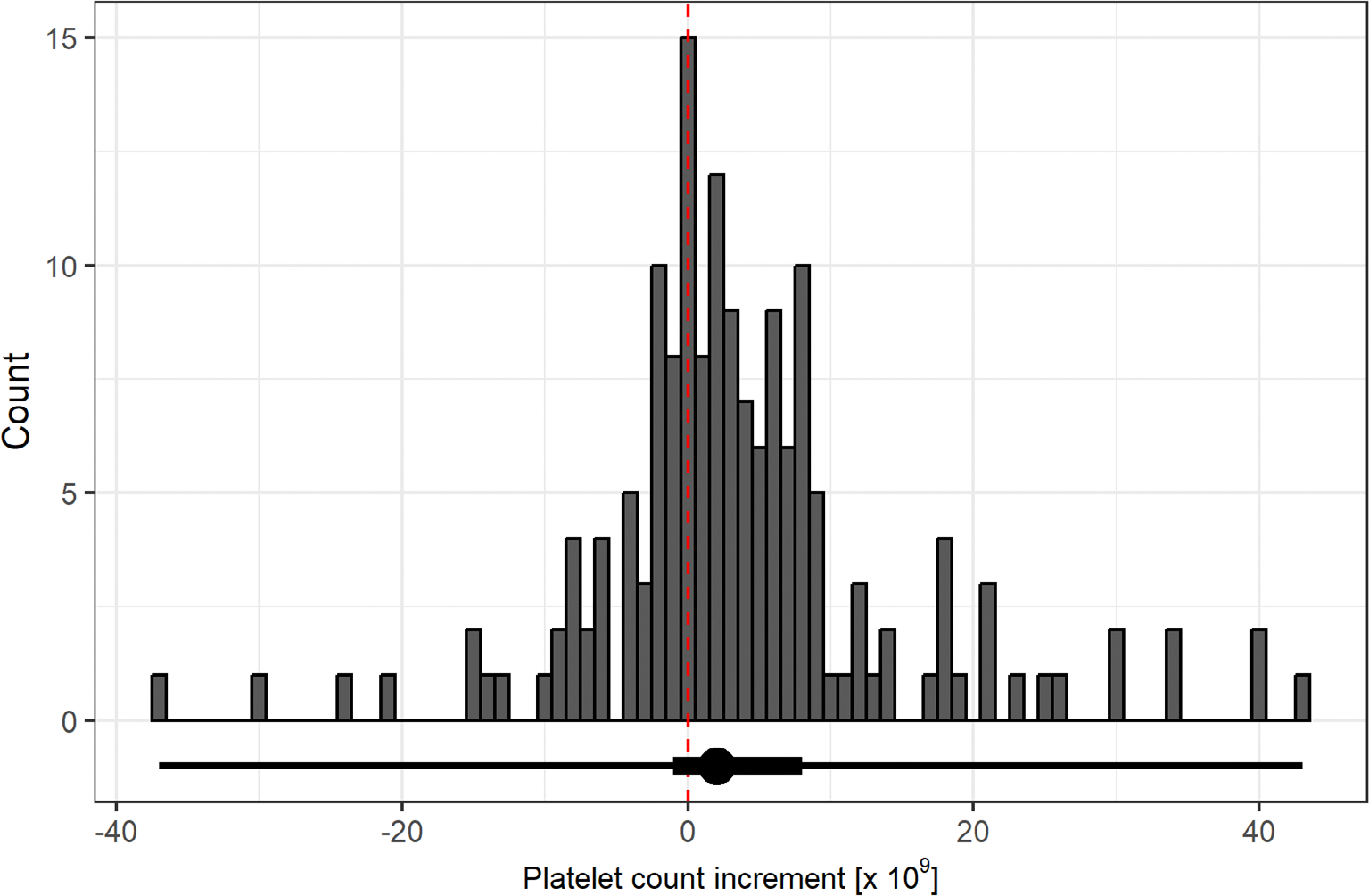
Platelet count increments for prophylactic transfusions. Distribution of platelet increments for 161 prophylactic transfusions used as single transfusions in ICU. Below the histogram is a horizontal box plot: the dot represents the median, the thick line represents the interquartile range and the thin line represents the range. The dashed red line marks a platelet increment of 0. Platelet increments could not be calculated for 22/183 (12.0%) prophylactic platelet transfusions (details in Data S1). ICU, intensive care unit.

The overall median platelet increment was 2 (IQR −1 to 8) × 10^9^/L (Figure 1), varying from 1 to 8 × 10^9^/L across countries (eFigure 7 in Data S1). The median pre-transfusion platelet count for transfusions with data on increment was 14 (IQR 7–26) × 10^9^/L. Platelet increments could not be computed for 22/183 (12%) transfusions. Sensitivity analysis excluding transfusions in proximity to bleeding or surgery and those used in patients with presumed hypo-proliferative thrombocytopenia (73.3%) produced similar increments of 2 (IQR −1 to 7) × 10^9^/L and 3 (−2 to 8) × 10^9^/L respectively, with median pre-transfusion platelet counts of 13 (7–24) × 10^9^/L and 30 (20–44) × 10^9^/L, respectively.

#### 3.2.4 | Timing of platelet transfusions

**FIGURE 2:**
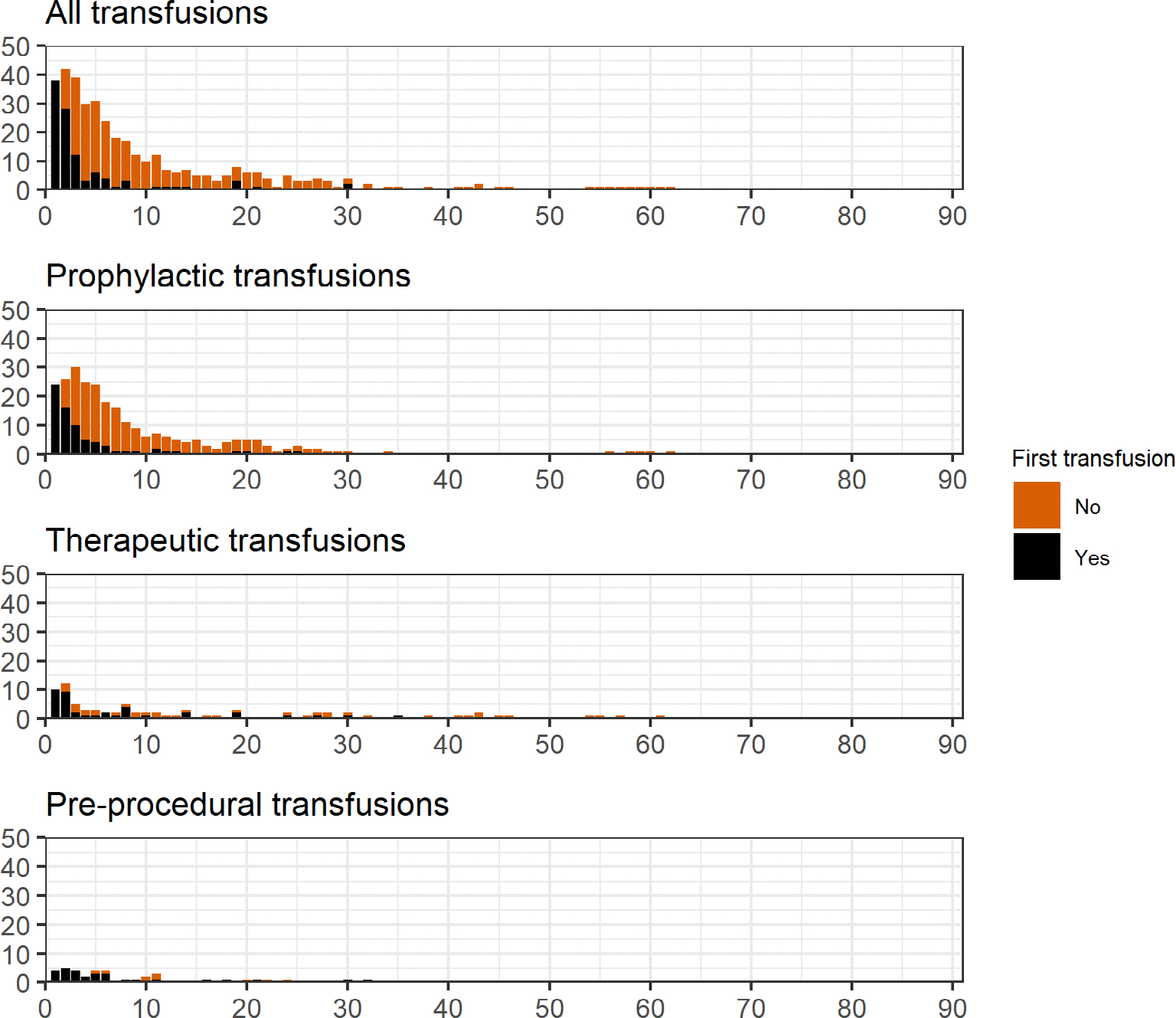
Number of patients receiving platelet transfusions in ICU according to days since ICU admission. Data are presented for all platelet transfusions and stratified by indication. Day 1 represents the day of ICU admission. The black part of each bar represents patients who received their first platelet transfusions on this day, and the orange part represents patients who received a platelet transfusion on this day but previously had received another. An identical figure is presented in eFigure 8 in Data S1 including transfusions used in operating rooms. ICU, intensive care unit.

**FIGURE 3:**
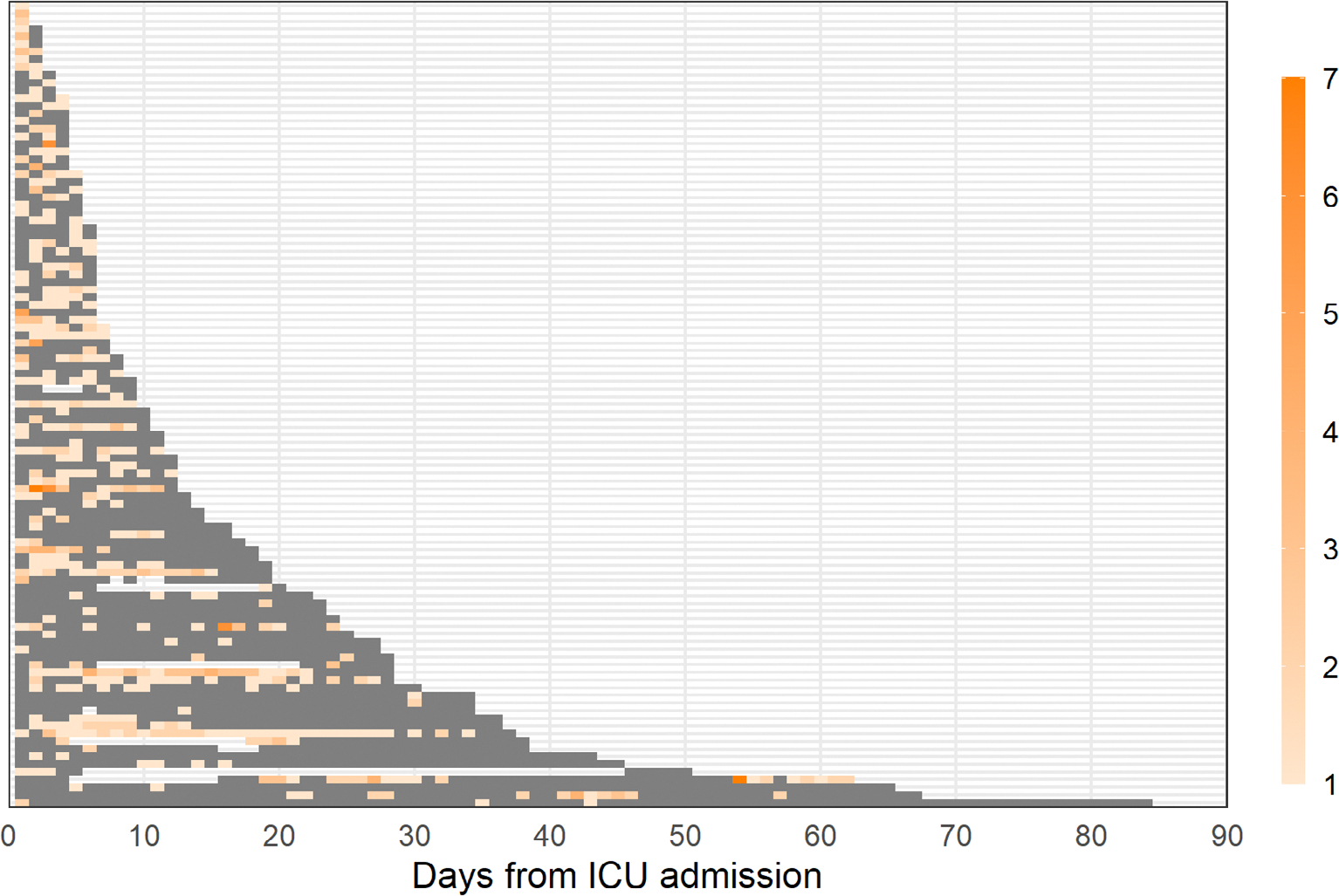
Timing and number of platelet transfusions in ICU. Timing and number of platelet transfusions used amongst the 105 patients who received platelet transfusion in ICU. Each horizontal line of tiles represents a patient, the grey-coloured tiles represent days in ICU where no platelets were transfused and the orange-coloured tiles represent days in ICU where the patient was transfused with platelets. The colour intensity corresponds to the number of platelet transfusions received that day. The median number of days with platelet transfusion was 2 (IQR 1–4) per patient and number of transfusions administered on these days was 1 (1–2). Similar figures for each indication and including transfusions used in operating rooms are presented in eFigures 9–12 in Data S1. ICU, intensive care unit.

The number of patients receiving platelet transfusion in ICU according to days from admission is presented in Figure 2. The median time to first transfusion was 2 (IQR 1–4) days (day 1 representing admission) and 82.9% and 90.5% of patients received their first transfusion within the first 5 and 10 days respectively. The timing and quantity of platelet transfusions used each day are presented in Figure 3 and stratified by indications in eFigures 9–11 in Data S1. Among the 105 patients receiving platelet transfusion in ICU, the median number of days with platelet transfusion was 2 (IQR 1–4) and the number of transfusions administered on these days was 1 (1–2). Sensitivity analyses including transfusions in operating rooms produced similar results (eFigures 8 and 12 in Data S1).

#### 3.2.5 | Platelet dosing

In total, 42/43 (97.7%) of centres responded to the survey (full results in Data S1). Most centres employed fixed dosing (73.8%) with reported median doses for pooled and apheresis products both ranging from 2.0 to 3.5 × 10^11^ platelets. Centres in France exclusively employed weight-based dosing with reported targeted doses of 0.5 to 0.7 × 10^11^ platelets per 10 kg actual body weight. A centre in Spain, reported using a single pooled product containing a median of 3.5 × 10^11^ platelets for patients with an actual body weight ≤90 kg and 2 for patients weighing >90 kg.

### 3.3 | Secondary clinical outcomes

Secondary clinical outcomes are presented in Table 2. Across strata, we observed numerically lower mortality among patients without severe thrombocytopenia receiving platelet transfusion despite having numerically higher rates of major bleeding, thrombosis and other transfusions. The numerically highest mortality was observed in patients with severe thrombocytopenia receiving platelet transfusion.

## 4 | DISCUSSION

In this sub-study of 504 acutely admitted adult ICU patients with thrombocytopenia from the PLOT-ICU study, 20.8% received platelet transfusion in ICU; 61.0% received pooled products, 21.9% received apheresis products and 17.1% received both. We observed variations between and within countries in type of platelet product, volumes and doses. Most centres used fixed dosing while use of weight-based dosing was primarily observed in France. Platelet increments following a single prophylactic platelet transfusion were limited. Outcomes in patients with and without severe thrombocytopenia who did and did not receive platelet transfusions varied.

In this study, most ICU patients received pooled platelet products. Denmark, Germany, Norway, Spain, Sweden and United Kingdom predominantly used pooled products while the United States and France mainly used apheresis products. To our knowledge, this variation has not been reported previously in international cohorts of ICU patients. However, varying use of pooled products has been reported previously across studies from centres in Canada, France and Germany,^3,26,30^ and in a survey among transfusion medicine experts from Europe, United States, Asia and Australasia,^22^ which aligns with our results.

A considerable number of patients were transfused with both products indicating that interchangeable use is common. Evidence from randomised clinical trials (RCTs) comparing pooled whole-blood-derived and apheresis-derived platelet transfusions is limited and primarily obtained in the haemato-oncologic setting.^16^ Although apheresis products may result in higher platelet increments and lower donor exposure compared to pooled products, this has not translated into clinical benefits^15–17^ and in the absence of firm evidence, many regard the two products as clinically equivalent.^17,31^ Interchangeable use is, therefore, likely to continue, primarily at the discretion of local transfusion services and available stock.

We observed considerable variation between countries in platelet transfusion volumes and doses. Determining the optimal platelet dose remains a major challenge in transfusion medicine with the two largest trials assessing prophylactic platelet transfusions in haematological patients reaching different conclusions. One trial was stopped early due to some WHO grade 4 bleedings occurring in the low dose arm (low dose: 1.5–3.0 × 10^11^/product, high dose: 3.0–6.0 × 10^11^/product)^18^ while the other found no difference between the low-, medium- and high dose arms (1.1 × 10^11^, 2.2 × 10^11^ or 4.4 × 10^11^/m^2^ body surface area).^19^ A meta-analysis found no statistically significant differences in bleeding outcomes or mortality between low-, medium- and high dose strategies (1.1 × 10^11^, 2.2 × 10^11^ or 4.4 × 10^11^/m^2^ body surface area) in haematological patients, however, higher doses led to fewer transfusions, but also a higher total dose transfused and more transfusion-related adverse events.^20^ Applying this evidence to ICU patients is challenging due to the presence of multiple underlying causes of thrombocytopenia^32^ and the simultaneous occurrence of various coagulation abnormalities.^33^

In addition to the limited evidence regarding type and doses of platelet transfusions, there may be other explanations for the observed variations in our study. First, numerous donor factors may impact product characteristics introducing biological variation.^34,35^ Second, divergent regulatory frameworks^21,36^ and organisation of blood transfusion services coupled with diverse manufacturing method adds additional variability.^21,22^

The observed platelet increment after a single prophylactic platelet transfusion in our study contrasts results from a large, retrospective, Canadian, multicentre study, reporting considerably higher platelet increments (23 [IQR 7–44] × 10^9^/L).^37^ There may be several explanations for this difference: first, the study population differed from ours in key aspects: 90% were surgical patients (primarily cardiac surgery), only 6% had sepsis and patients with cancer or chemotherapy-induced thrombocytopenia were excluded.^37^ In our study, most single prophylactic platelet transfusions were used in patients with presumed hypoproliferative thrombocytopenia who may be prone to low increments. Indeed, studies with high proportions of critically ill patients with presumed hypo-proliferative thrombocytopenia have reported poor platelet increments in approximately 50%–75% of prophylactic platelet transfusions^26,27^ and haematological malignancy has been associated with poor transfusion response.^27^ Second, pre-transfusion platelet counts were markedly higher than those observed in our study, suggesting that most platelet transfusions were likely administered for reasons other than prophylaxis.^37^ Third, the post-transfusion platelet count was defined as the closest platelet count measured within 4–24 h following transfusion (median of 7 h).^37^ In the original PLOT-ICU study, data collection was limited to calendar days and the exact timing between transfusion and subsequent platelet count measurement was not accrued.^4^ Consequently, we assumed that the lowest platelet count the following day represented the post-transfusion platelet count.^23^ Since time from transfusion to platelet count measurement may impact platelet increment, there is a risk that the increment, however short-lived, remained undetected in our study.^38,39^

Platelet transfusion is not without risks as demonstrated by RCTs in preterm infants^40^ and patients with intracerebral haemorrhage receiving antiplatelet therapy^41^ and various observational studies encompassing both cancer patients^42^ and ICU patients.^43–45^ We observed numerically lower mortality despite higher rates of major bleeding, thrombosis and other transfusions in patients without severe thrombocytopenia receiving platelet transfusion compared with the other strata. This observation is likely attributable to most of these patients being admitted after surgery with haemorrhage. In contrast, we observed numerically higher mortality among patients with severe thrombocytopenia receiving platelet transfusions. These results should be interpreted cautiously due to potential confounding by indication and viewed as hypothesis-generating only. Evidence from observational studies regarding the effect of platelet transfusions on mortality in ICU patients is conflicting.^45,46^

The data presented in this study are important to researchers and trialists as they highlight current platelet transfusion practice and areas of discrepancies where trials are needed. Moreover, we report outcomes in severely thrombocytopenic patients receiving platelet transfusion in the ICU which are likely to represent the target population in future platelet transfusion trials in the ICU setting.

The strengths of this study reflect those of the PLOT-ICU study and include the large, international ICU population, prospective data collection and the quality and completeness of the data. Although the study was planned post hoc, and should be considered exploratory, it was conducted according to a published protocol, which increases transparency and reliability.^47,48^

The study also has limitations. Our sample size of 565 platelet transfusions may be insufficient to accurately describe platelet transfusion practices and product characteristics across countries; some countries were only represented by few centres and may not reflect national practice. The data collection in the PLOT-ICU study focused on events in ICU, with limited information events occurring intraoperatively and our results may not apply outside the ICU setting.^4^ Additionally, platelet transfusion volumes were collected as daily totals for each indication by each product type,^4^ and computed volumes on days with multiple transfusions may not reflect actual volumes. Additionally, many ICUs (especially those using fixed dosing) registered average platelet transfusion volumes rather than exact volumes in the medical records which adds to the uncertainty. Finally, the structure of the PLOT-ICU database did not allow us to assess the timing of platelet transfusion and subsequent platelet count measurements and were not able to assess corrected platelet count increments (corrected for patient size and platelet dose) because exact platelet dose contained in the individual platelet products was not available.

## 5 | CONCLUSIONS

In this sub-study of 504 acutely admitted adult thrombocytopenic ICU patients from the PLOT-ICU study, 20.8% received platelet transfusion in ICU; 61.0% received pooled products, 21.9% received apheresis products and 17.1% received both. We observed considerable variation in product types, volumes, and doses utilised between and within countries, and the platelet increment following a single prophylactic platelet transfusion was limited.

## Supplementary Material

Supplement

## Figures and Tables

**Table 1:** Baseline characteristics.

	Non-severe thrombocytopenia		Severe thrombocytopenia	
	Not receiving platelet transfusion in ICU (*n* = 352)^a^	Receiving platelet transfusion in ICU (*n* = 18)^b^	Not receiving platelet transfusion in ICU (*n* = 47)	Receiving platelet transfusion in ICU (*n* = 87)^c^
Age [years]	64 (52–74)	63 (57–69)	61 (47–70)	59 (51–68)
Female sex	115 (32.7%)	6 (33.3%)	16 (34.0%)	33 (37.9%)
**Comorbidities**				
Pulmonary disease	43 (12.2%)	1 (5.6%)	10 (21.3%)	10 (11.5%)
Ischaemic heart disease or heart failure	64 (18.2%)	3 (16.7%)	9 (19.1%)	6 (6.9%)
Chronic renal failure	40 (11.4%)	1 (5.6%)	7 (14.9%)	6 (6.9%)
Chronic liver failure	29 (8.2%)	2 (11.1%)	8 (17.0%)	11 (12.6%)
Solid tumour cancer	53 (15.1%)	4 (22.2%)	6 (12.8%)	10 (11.5%)
Haematological malignancy	17 (4.8%)	2 (11.1%)	13 (27.7%)	43 (49.4%)
Immunosuppression^d^	26 (7.4%)	0 (0.0%)	4 (8.5%)	12 (13.8%)
Previous thrombo-embolism	44 (12.5%)	4 (22.2%)	6 (12.8%)	7 (8.0%)
**Admission characteristics**				
Days hospitalised prior to ICU admission	2 (1–3)	2 (1–3)	2 (1–5)	9 (2–18)
Surgery during hospitalisation	101 (28.7%)	10 (55.6%)	5 (10.6%)	21 (24.1%)
Source of admission				
Emergency department	168 (47.7%)	3 (16.7%)	24 (51.1%)	16 (18.4%)
Hospital ward	93 (26.4%)	5 (27.8%)	20 (42.6%)	52 (59.8%)
OR/recovery room	58 (16.5%)	8 (44.4%)	1 (2.1%)	10 (11.5%)
Another ICU	33 (9.4%)	2 (11.1%)	2 (4.3%)	9 (10.3%)
Reason for ICU admission				
Circulatory	101 (28.7%)	5 (27.8%)	15 (31.9%)	32 (36.8%)
Haemorrhage	23 (6.5%)	7 (38.9%)	2 (4.3%)	4 (4.6%)
Neurological	56 (15.9%	1 (5.6%)	3 (6.4%)	7 (8.0%)
Respiratory	91 (25.9%)	1 (5.6%)	13 (27.7%)	24 (27.6%)
Trauma	19 (5.4%)	3 (16.7%)	0 (0.0%)	0 (0.0%)
Other	62 (17.6%)	1 (5.6%)	14 (29.8%)	20 (23.0%)
Septic shock	67 (19.0%)	2 (11.1%)	19 (40.4%)	28 (32.2%)
Acute liver failure	17 (4.8%)	1 (5.6%)	4 (8.5%)	12 (13.8%)
Any WHO bleeding^e^	68 (19.3%)	9 (50.0%)	5 (10.6%)	14 (16.1%)
SMS-ICU^f^	17 (13–22)	21 (19–24)	20 (15–24)	20 (15–24)
**Treatments before ICU admission**				
Stem cell transplantation^g^	3 (0.9%)	1 (5.6%)	4 (8.5%)	20 (23.0%)
Chemotherapy^h^	22 (6.2%)	2 (11.1%)	9 (19.1%)	26 (29.9%)
Anticoagulation^i^	131 (37.2%)	6 (33.3%)	10 (21.3%)	20 (23.0%)
Platelet inhibitors^i^	54 (15.3%)	5 (27.8%)	2 (4.3%)	5 (5.7%)
Platelet transfusion^j^	17 (4.8%)	3 (16.7%)	3 (6.4%)	27 (31.0%)
**Biochemistry at ICU admission**				
Platelet count [×10^9^/L]^k^	159 (123–210)	161 (119–239)	63 (39–136)	47 (20–122)
Haemoglobin [g/dL]^l^	11.9 (9.9–14.1)	8.9 (8.1–12.4)	10.8 (8.2–12.3)	9.0 (7.9–11.0)
INR > 1.5^m^	51 (19.1%)	3 (21.4%)	11 (29.7%)	18 (25.0%)

*Note:* Characteristics at ICU admission stratified on platelet transfusion status in ICU and severe thrombocytopenia (<50 × 10^9^/L). Categorical data are presented as numbers and percentages and continuous data as medians with interquartile ranges. Full definitions are available elsewhere^4,25^ and in Data S1.

Abbreviations: ICU, intensive care unit; INR, international normalised ratio; OR, operating room; SMS-ICU, Simplified Mortality Score for the Intensive Care Unit; WHO, World Health Organization.

a Nine patients (2.6%) with non-severe thrombocytopenia not receiving platelet transfusions in ICU received 18 platelet transfusions in operating rooms during ICU admission.

b Three patients (16.7%) with non-severe thrombocytopenia who received platelet transfusions in ICU also received 8 platelet transfusions in operating theatres during ICU admission.

c Fourteen patients (16.1%) with severe thrombocytopenia who received platelet transfusions in ICU also received 27 platelet transfusions in operating rooms during ICU admission.

d Immunosuppression not related to AIDS or cancer including solid organ transplant and conditions requiring long-term (>30 days) or high dose (>1 mg/kg/ day) treatment with steroids, or any immunosuppressive drug for more than 30 days.

e Grade 1–4 bleeding within 24 h before ICU admission.

f Illness severity score ranging from 0 to 42 with corresponding predicted 90-day mortality of 3.3%-91.0%.^28,29^ Details in Data S1.

g Allogenic or autologous stem cell transplantation within 1 year before to ICU admission.

h Within 6 weeks before ICU admission.

i Any dose within 48 h before ICU admission.

j Within 24 h before ICU admission.

k Baseline platelet counts were missing in 53 (10.5%) patients.

l Baseline haemoglobin values were missing in 34 (6.7%) patients.

m Baseline INR values were missing in 114 (22.6%) patients.

**TABLE 2:** Secondary clinical outcomes.

	Non-severe thrombocytopenia		Severe thrombocytopenia	
	Not receiving platelet transfusion in ICU (*n* = 352)^a^	Receiving platelet transfusion in ICU (*n* = 18)^b^	Not receiving platelet transfusion in ICU (*n* = 47)^a^	Receiving platelet transfusion in ICU (*n* = 87)^c^
90-day mortality^d^	101 (28.8%)	4 (22.2%)	22 (46.8%)	47 (54.0%)
Major bleeding in ICU^e^	34 (9.7%)	13 (72.2%)	6 (12.8%)	27 (31.0%)
New thrombosis in ICU	27 (7.7%)	4 (22.2%)	2 (4.3%)	5 (5.7%)
RBC transfusions^f^	115 (32.7%)	15 (83.3%)	17 (36.2%)	70 (80.5%)
Plasma transfusion^g^	54 (15.3%)	14 (77.8%)	6 (12.8%)	27 (31.0%)

*Note:* Secondary clinical outcomes stratified by platelet transfusion status in ICU and severe thrombocytopenia (<50 × 10^9^/L). Categorical data are presented as numbers and percentages. Full outcome definitions are available elsewhere^4,25^ and in Data S1.

Abbreviations: ICU, intensive care unit; RBC, red blood cell.

a Nine patients (2.6%) with non-severe thrombocytopenia not receiving platelet transfusions in ICU received 18 platelet transfusions in operating rooms during ICU admission.

b Three patients (16.7%) with non-severe thrombocytopenia who received platelet transfusions in ICU also received 8 platelet transfusions in operating rooms during ICU admission.

c Fourteen patients (16.1%) with severe thrombocytopenia who received platelet transfusions in ICU also received 27 platelet transfusions in operating theatres during ICU admission.

d One patient (0.2%) had missing data for 90-day mortality.

e Bleeding of modified World Health Organization grade 3 or 4.

f At least one red blood cell (RBC) transfusion during ICU admission (transfusions in operating theatres included).

g At least one transfusion with plasma products during ICU admission (transfusions in operating theatres included). Plasma products included fresh frozen plasma, cryoprecipitate, cryo-depleted plasma and octaplasLG^®^/octaplas^®^.

## Data Availability

A de-identified dataset may be shared with other researchers upon reasonable request (i.e. a research proposal outlining objectives, methodologies and plans for data usage) and approval by the PLOT-ICU Steering Committee. Prior to the release of the data, concerned parties must sign appropriate agreements outlining terms and conditions, confidentiality measures, other considerations (e.g. ethical, and legal requirements) and confirm that the data will be used for the agreed purpose only.
