# Supplementary material for: Platelet transfusions in adult ICU patients with thrombocytopenia: A sub-study of the PLOT-ICU inception cohort study
Source: Acta Anaesthesiol Scand. Author manuscript; Available in PMC 2025 Jul 1. (PMC11729610; doi:10.1111/aas.14467)
Supplement: Supplement [file NIHMS2043753-supplement-Supplement.docx]

Platelet transfusions in adult ICU patients with thrombocytopenia: a sub-study of the PLOT-ICU inception cohort study

SUPPLEMENT

Anthon CT, Pène F, Perner A, Azoulay E, Puxty K, van De Louw A, Chawla S, Castro P, Povoa P, Coelho L, Metaxa V, Kochanek M, Liebregts T, Kander T, Sivula M, Andreasen JB, Nielsen LB, Hvas CL, Dufranc E, Canet E, Wright CJ, Schmidt J, Uhel F, Missri L, Krag M, Cos Badia E, Díaz-Lagares C, Menat S, Voiriot G, Clausen NE, Lorentzen K, Kvåle R, Barratt-Due A, Hildebrandt T, Holten AR, Strand K, Bestle MH, Klepstad P, Vimpere D, Paulino C, Lueck C, Juhl CS, Costa C, Bådstøløkken PM, Lêdo LSA, Møller, MH, Russell L.

**Contents**

[**Abbreviations** 1](#_Toc161138627)

[**Additions to the protocol** 2](#_Toc161138628)

[**STROBE checklist** 3](#_Toc161138629)

[**Variable definitions** 5](#_Toc161138630)

[**SMS-ICU** 9](#_Toc161138631)

[**Modified WHO bleeding classification** 10](#_Toc161138632)

[**Outcomes and reporting** 11](#_Toc161138633)

[**Missing data** 13](#_Toc161138634)

[**Platelet transfusion used in operating rooms** 14](#_Toc161138635)

[**eTable 1: Primary outcome stratified on country** 15](#_Toc161138636)

[**eFigure 1. Type of platelet transfusion used in ICU across countries** 16](#_Toc161138637)

[**eFigure 2. Type of platelet transfusion used in ICU across sites within countries** 17](#_Toc161138638)

[**eFigure 3. Relative distributions of volumes for platelet transfusions used in ICU across countries** 18](#_Toc161138639)

[**eFigure 4. Relative distributions of volumes for platelet transfusions used in ICU across sites** 19](#_Toc161138640)

[**eFigure 5. Relative distributions of volumes for platelet transfusions used in ICU and operating rooms across countries** 20](#_Toc161138641)

[**eFigure 6. Relative distributions of volumes for platelet transfusions used in ICU and operating rooms across sites** 21](#_Toc161138642)

[**eFigure 7. Platelet count increments across countries** 22](#_Toc161138643)

[**eFigure 8. Number of patients receiving platelet transfusion in ICU or operating rooms according to days from ICU admission** 23](#_Toc161138644)

[**eFigure 9. Timing and number of prophylactic platelet transfusions in ICU** 24](#_Toc161138645)

[**eFigure 10. Timing and number of therapeutic platelet transfusions in ICU** 25](#_Toc161138646)

[**eFigure 11. Timing and number of pre-procedural platelet transfusions in ICU.** 26](#_Toc161138647)

[**eFigure 12. Timing and number of platelet transfusions in ICU or operating rooms** 27](#_Toc161138648)

[**Additional survey results** 28](#_Toc161138649)

[**eTable 2: Overview of platelet dosing** 29](#_Toc161138650)

[**References** 30](#_Toc161138651)

# **Abbreviations**

DEN Denmark

ESP Spain

FIN Finland

FRA France

GBR United Kingdom

GER Germany

ICU Intensive care unit

IQR Interquartile range

NOR Norway

POR Portugal

SMS-ICU Simplified Mortality Score for the Intensive Care Unit

STROBE The Strengthening the Reporting of Observational Studies in Epidemiology Statement: guidelines for reporting observational studies.

SWE Sweden

USA United States

WHO World Health Organization

# **Additions to the protocol**

| **Additions** | | **Motivation** |
| --- | --- | --- |
| 1 | We added the number of patients receiving both types of platelet products (i.e., at least one pooled whole-blood-derived and at least one apheresis-derived platelet transfusion) to the primary outcome. | This outcome nuanced the description of current practice and highlighted the interchangeable use of pooled and apheresis products. |
| 2 | We added the number of platelet transfusion administered in ICU categorised by product type (i.e., number of pooled whole-blood-derived and apheresis-derived platelet transfusion administered) overall, across countries, and across sites. | This provided an overview of the use of pooled and apheresis products overall, between- and within countries and highlighted between- and within country variation in practice. |
| 3 | We added stratification on sites in the analysis of platelet transfusions volumes. | This analysis nuances the description of variation in platelet transfusions volumes and highlighted within country variation. |
| 4 | We added sensitivity analyses including platelet transfusions used in operating rooms in the analyses of platelet transfusion volumes and timing of platelet transfusion. | These sensitivity analyses comprise all available data and extents the interpretation of the analyses to all platelet transfusions administered during ICU stay including those administered in operating rooms. |
| 5 | We added a sensitivity analysis excluding platelet transfusions administered to patients with presumed hypo-proliferative thrombocytopenia (defined as haematological malignancy and/or treatment with hematopoietic stem cell transplantation and/or chemotherapy) to the analysis of post-transfusion platelet increment for single prophylactic platelet transfusions. | This sensitivity analyses challenged the results of the primary analyses. This was done as patients with presumed hypo-proliferative received most prophylactic platelet transfusions and as these patients may have particularly low platelet increments which could impact the overall results. |

# **STROBE checklist**

|  | **Item No** | **Recommendation** | **Page  No** |
| --- | --- | --- | --- |
| Title and abstract | 1 | (*a*) Indicate the study’s design with a commonly used term in the title or the abstract | 1 |
|  |  | (*b*) Provide in the abstract an informative and balanced summary of what was done and what was found | 7 |
| **Introduction** | | | |
| Background/rationale | 2 | Explain the scientific background and rationale for the investigation being reported | 8 |
| Objectives | 3 | State specific objectives, including any prespecified hypotheses | 8 |
| **Methods** | | | |
| Study design | 4 | Present key elements of study design early in the paper | 9 |
| Setting | 5 | Describe the setting, locations, and relevant dates, including periods of recruitment, exposure, follow-up, and data collection | 9-10 |
| Participants | 6 | (*a*) *Cohort study*—Give the eligibility criteria, and the sources and methods of selection of participants. Describe methods of follow-up  *Case-control study*—Give the eligibility criteria, and the sources and methods of case ascertainment and control selection. Give the rationale for the choice of cases and controls  *Cross-sectional study*—Give the eligibility criteria, and the sources and methods of selection of participants | 9 |
|  |  | (*b*) *Cohort study*—For matched studies, give matching criteria and number of exposed and unexposed  *Case-control study*—For matched studies, give matching criteria and the number of controls per case | NA |
| Variables | 7 | Clearly define all outcomes, exposures, predictors, potential confounders, and effect modifiers. Give diagnostic criteria, if applicable | 9-10, suppl. 11-12 |
| Data sources/ measurement | 8* | For each variable of interest, give sources of data and details of methods of assessment (measurement). Describe comparability of assessment methods if there is more than one group | suppl. 5-8 |
| Bias | 9 | Describe any efforts to address potential sources of bias | NA |
| Study size | 10 | Explain how the study size was arrived at | 10 |
| Quantitative variables | 11 | Explain how quantitative variables were handled in the analyses. If applicable, describe which groupings were chosen and why | 10 |
| Statistical methods | 12 | (*a*) Describe all statistical methods, including those used to control for confounding | 10 |
|  |  | (*b*) Describe any methods used to examine subgroups and interactions | NA |
|  |  | (*c*) Explain how missing data were addressed | 11, suppl.13 |
|  |  | (*d*) *Cohort study*—If applicable, explain how loss to follow-up was addressed  *Case-control study*—If applicable, explain how matching of cases and controls was addressed  *Cross-sectional study*—If applicable, describe analytical methods taking account of sampling strategy | 11,  suppl. 13 |
|  |  | (*e*) Describe any sensitivity analyses | 10-11 |
| Results | | | |
| Participants | 13* | (a) Report numbers of individuals at each stage of study—eg numbers potentially eligible, examined for eligibility, confirmed eligible, included in the study, completing follow-up, and analysed | 12 |
|  |  | (b) Give reasons for non-participation at each stage | NA |
|  |  | (c) Consider use of a flow diagram | NA |
| Descriptive data | 14* | (a) Give characteristics of study participants (eg demographic, clinical, social) and information on exposures and potential confounders | 12 and Table 1 |
|  |  | (b) Indicate number of participants with missing data for each variable of interest | Table 1 |
|  |  | (c) *Cohort study*—Summarise follow-up time (eg, average and total amount) | NA |
| Outcome data | 15* | *Cohort study*—Report numbers of outcome events or summary measures over time | 12-13 |
|  |  | *Case-control study—*Report numbers in each exposure category, or summary measures of exposure | Table 2 |
|  |  | *Cross-sectional study—*Report numbers of outcome events or summary measures | NA |
| Main results | 16 | (*a*) Give unadjusted estimates and, if applicable, confounder-adjusted estimates and their precision (eg, 95% confidence interval). Make clear which confounders were adjusted for and why they were included | 12 |
|  |  | (*b*) Report category boundaries when continuous variables were categorized | NA |
|  |  | (*c*) If relevant, consider translating estimates of relative risk into absolute risk for a meaningful time period | NA |
| Other analyses | 17 | Report other analyses done—eg analyses of subgroups and interactions, and sensitivity analyses | 12-13 |
| Discussion | | | |
| Key results | 18 | Summarise key results with reference to study objectives | 14 |
| Limitations | 19 | Discuss limitations of the study, taking into account sources of potential bias or imprecision. Discuss both direction and magnitude of any potential bias | 15 |
| Interpretation | 20 | Give a cautious overall interpretation of results considering objectives, limitations, multiplicity of analyses, results from similar studies, and other relevant evidence | 16 |
| Generalisability | 21 | Discuss the generalisability (external validity) of the study results | 16 |
| Other information | | | |
| Funding | 22 | Give the source of funding and the role of the funders for the present study and, if applicable, for the original study on which the present article is based | 6 |

*Give information separately for cases and controls in case-control studies and, if applicable, for exposed and unexposed groups in cohort and cross-sectional studies.

**Note:** An Explanation and Elaboration article discusses each checklist item and gives methodological background and published examples of transparent reporting. The STROBE checklist is best used in conjunction with this article (freely available on the Web sites of PLoS Medicine at http://www.plosmedicine.org/, Annals of Internal Medicine at http://www.annals.org/, and Epidemiology at http://www.epidem.com/). Information on the STROBE Initiative is available at [www.strobe-statement.org](http://www.strobe-statement.org) [1, 2].

# **Variable definitions**

The PLOT-ICU study collected routinely available data from medical records [3, 4].

**Baseline variables**

- Age: age at ICU admission (whole years)
- Sex: genotypic sex
- Chronic pulmonary disease: treatment at time of hospital admission with any relevant drug indicating chronic pulmonary disease (e.g., COPD, asthma) e.g., albuterol, levalbuterol, salmeterol, formoterol, arformoterol, indacaterol, vilanterol, olodaterol, tiotropium, aclidinium, umeclidinium, glycopyrronium, budesonide and fluticasone
- Ischaemic heart disease or heart failure: previous myocardial infarction, invasive intervention for coronary artery disease, stable or unstable angina, NYHA class 3 or 4 or measured LVEF < 40%
- Chronic renal failure: need for chronic renal support including continuous or intermittent renal replacement therapy or S-creatinine > 3.6 g/dL / 300 μmol/L before hospital admission
- Chronic liver failure: portal hypertension, cirrhosis (proven by biopsy, CT scan or ultrasound), history of variceal bleeding or hepatic encephalopathy
- Haematological malignancy: acute lymphoblastic leukaemia, acute myelogenous leukaemia, chronic lymphocytic leukaemia, chronic myelogenous leukaemia, t-cell prolymphocytic leukaemia, b-cell prolymphocytic leukaemia, large granular lymphocytic leukaemia, lymphomas including Hodgkin's lymphoma and non-Hodgkin lymphoma (e.g., small lymphocytic lymphoma, lymphoblastic lymphoma, diffuse large B-cell lymphoma, follicular lymphoma, mantle cell lymphoma, Hairy cell leukaemia, Marginal zone lymphoma, Burkitt's lymphoma, Post-transplant lymphoproliferative disorder, Waldenstöm's macroglobulinemia, natural killer- or T-cell lymphomas), multiple myeloma/plasma cell myeloma, myelodysplastic syndrome, other myeloproliferative neoplasms including chronic neutrophilic leukaemia, primary myelofibrosis and mast cell disease
- Solid tumour cancer: any solid non-haematological malignant tumour confirmed by surgery, CT scan or any other method
- Immunosuppression: immunosuppression not related to cancer or AIDS including solid organ transplant or conditions requiring long-term (> 30 days) or high-dose (> 1 mg/kg/day) steroids, or any immunosuppressive drug for more than 30 days
- Previous thrombo-embolism: history of arterial or venous thromboembolism including peripheral arterial thrombosis, acute coronary thrombosis, acute mesenteric ischemia, acute ischaemic stroke, central vein thrombosis, pulmonary embolus, peripheral venous thrombosis, and cerebral vein thrombosis (see below for details)
- Days spent in hospital prior to ICU admission: number of days in hospital prior to ICU admission calculated as the difference in days between the dates of hospital and ICU admission
- Surgery: Surgery (acute or elective) during the current hospitalisation
- Primary reason for ICU admission: neurological, respiratory, circulatory, trauma, haemorrhage, other according to the attending physician or documented in patient records
- Source of ICU admission: emergency department (any accident/emergency/casualty/acute department or directly from the pre-hospital setting via ambulance service or similar), general ward, operating theatre, or recovery room (any surgical theatre, endoscopy and angiography suite or recovery facilities observing post-operative patients), another ICU
- Haematopoietic stem cell transplantation: autologous or allogenic haematopoietic stem cell transplantation within 1 year before ICU admission
- Chemotherapy: treatment with chemotherapy including Bortezomib, Carboplatin, Cisplatin, Cyclophosphamide, Dacarbazine, Docetaxel, Doxorubicin, Etoposide, Fluorouracil, Gemcitabine, Hydroxycarbamide, Ibritumomab tiuxetan, Ifosfamide, Irinotecan, Leucovorin, Methotrexate, Oxaliplatin, Panobinostat, Temozolomide, Tamoxifen, Vincristine within 6 weeks before ICU admission
- Anticoagulation: treatment with anticoagulating agents in any dose within 48 hours before ICU admission including unfractionated heparin, low-molecular-weight heparin, new oral anticoagulant drugs, vitamin K-antagonists and intravenous direct thrombin inhibitors
- Platelet inhibitors: treatment with platelet inhibitors within 48 hours before ICU admission including ADP-receptor inhibitors, acetylsalicylic acid, and dipyridamole
- Septic shock: suspected or confirmed site of infection or positive blood culture AND ongoing infusion of vasopressor/inotrope agent to maintain a mean arterial blood pressure of 65 mmHg or above AND lactate of 2 mmol/L or above in any plasma sample in the first 24 hours of ICU admission [5]
- Acute liver failure: severe liver injury, potentially reversible in nature and with onset of hepatic encephalopathy within eight weeks of the first symptoms (i.e., jaundice) in the absence of pre-existing liver disease [6]
- SMS-ICU: an illness severity score [0–42 points]. See below for details.
- Platelet count: latest platelet count within 24 before ICU admission (x10^9^/L)
- Haemoglobin: latest haemoglobin within 24 before ICU admission (g/L)
- INR: latest INR within 24 hours before ICU admission
- Bleeding: modified WHO grade 1-4 bleeding at ICU admission. See below for details.
- Platelet transfusions: platelet transfusions within 24 before ICU admission

**Daily variables**

- Lowest platelet count: lowest platelet count of the day (x10^9^/L)
- Number of apheresis-derived platelet transfusions: total number of apheresis derived platelet transfusions administered in ICU for each indication (see below) that day
- Volume of apheresis-derived platelet transfusions: total volume (mL) of apheresis derived platelet transfusions administered in ICU for each indication (see below) that day
- Number of pooled whole-blood-derived platelet transfusions: total number of pooled whole-blood-derived platelet transfusions administered in ICU for each indication (see below) that day
- Volume of pooled whole-blood-derived platelet transfusions: total volume (mL) of pooled whole-blood-derived platelet transfusions administered in ICU for each indication administered (see below) that day
- Indication for platelet transfusion used in ICU:
  - Prophylaxis was defined as any non-procedural platelet transfusion administered in the ICU to patients to prevent or reduce the risk of bleeding.
  - Pre-procedural was defined as any platelet transfusion administered in the ICU to prevent or reduce the risk of bleeding before an invasive procedure (e.g., central venous catheter or dialysis catheter placement/removal, lumbar puncture, epidural catheter placement/removal, biopsies, pigtail catheter placement/removal, chest tube placement/removal, CNS surgery (including placement of EVD or ICP device), percutaneous dilatational tracheostomy, or surgery of any type).
  - Therapeutic was defined as any platelet transfusion administered in the ICU specifically to treat to bleeding
- Platelet count prior to transfusion: latest platelet count prior to platelet transfusion for each indication (x10^9^/L). If two or more transfusions were administered for the same indication on the same day, only the highest platelet count prior to any of the transfusions for that indication was registered
- Number of platelet transfusions used in operating rooms: total number of platelet transfusions used in operating rooms that day
- Volume of platelet transfusions used in operating rooms: total volume (mL) of platelet transfusions used in operating rooms that day
- Number of RBC transfusions: total number of RBC transfusions used in the ICU or in operating rooms that day
- Volume of RBC transfusion: total volume (mL) of RBC transfusions used in the ICU or in operating rooms that day
- Plasma transfusions: total number of plasma transfusions used in the ICU or in operating rooms that day. Includes any type of plasma product (fresh frozen plasma, cryoprecipitate, cryo-depleted plasma and octaplasLG®)
- Volume of plasma transfusions: total volume (mL) of plasma transfusions used in the ICU or during surgery in the operating theatre that day including any type of plasma product (fresh frozen plasma, cryoprecipitate, cryo-depleted plasma and octaplasLG®)
- WHO grade 3 or 4 bleeding: bleeding graded as a modified WHO grade 3 or 4 that day (see below for details)
- New thrombotic event: any new thrombotic event including arterial or venous thromboembolism; peripheral arterial thrombosis, acute coronary thrombosis, acute mesenteric ischemia, acute ischaemic stroke, central vein thrombosis, pulmonary embolus, peripheral venous thrombosis, and cerebral vein thrombosis

**Follow-up variables**

- Death within 90 days: death within 90 of ICU admission (i.e., inclusion)

**Additional variables**

The following variables were collected from investigators at each site participating in the original PLOT-ICU study.

- Availability of pooled-whole-blood-derived platelet transfusions: availability of platelet transfusions derived from whole blood donations by pooling platelet concentrates from multiple donors
- Method of manufacturing for pooled-whole-blood-derived platelet transfusions: Platelet rich plasma method, buffy coat method, single centrifugation method
- Availability of apheresis derived platelet transfusions: availability of platelet transfusions derived from a single donor by plateletpheresis
- Fixed dosing: a (constant) platelet dose independent of the patient’s bodyweight
  - Average platelet dose contained in pooled-whole-blood-derived platelet transfusions: mean or median number of platelets contained in pooled-whole-blood-derived platelet transfusions issued by the relevant transfusion service
  - Average platelet dose contained in apheresis derived platelet transfusions: mean or median number of platelets contained in apheresis derived platelet transfusions issued by the relevant transfusion service
- Weight-based dosing: a (varying) platelet dose adjusted for the patient’s bodyweight
  - Target dose per kg body weight: the target number of platelets per kg bodyweight

# **SMS-ICU**

| **Total score and predicted 90-day mortality risk** | | | |
| --- | --- | --- | --- |
| 0 | 3.3% | 22 | 40.1% |
| 3 | 4.8% | 23 | 43.4% |
| 4 | 5.5% | 24 | 46.7% |
| 5 | 6.2% | 25 | 50.1% |
| 6 | 7.1% | 26 | 53.5% |
| 7 | 8.0% | 27 | 56.9% |
| 8 | 9.1% | 28 | 60.2% |
| 9 | 10.3% | 29 | 63.4% |
| 10 | 11.6% | 30 | 66.4% |
| 11 | 13.1% | 31 | 69.4% |
| 12 | 14.7% | 32 | 72.2% |
| 13 | 16.5% | 33 | 74.8% |
| 14 | 18.4% | 34 | 77.3% |
| 15 | 20.5% | 35 | 79.6% |
| 16 | 22.8% | 36 | 81.7% |
| 17 | 25.3% | 37 | 83.7% |
| 18 | 28.0% | 38 | 85.4% |
| 19 | 30.8% | 39 | 87.0% |
| 20 | 33.8% | 41 | 89.8% |
| 21 | 36.9% | 42 | 91.0% |

| **Variable** | **Points** |
| --- | --- |
| **Age** | |
| ≤ 39 years | 0 |
| 40 – 59 years | 5 |
| 60 – 79 years | 10 |
| ≥ 80 years | 13 |
| **Lowest systolic blood pressure** | |
| ≤ 49 mmHg | 6 |
| 50 – 69 mmHg | 5 |
| 70 – 89 mmHg | 3 |
| ≥ 90 mmHg | 0 |
| **Acute surgical admission** | |
| No | 3 |
| Yes | 0 |
| **Haematological malignancy or metastatic cancer** | |
| No | 0 |
| Yes | 7 |
| **Vasopressor/inotropes ^a^** | |
| No | 0 |
| Yes | 4 |
| **Respiratory support ^b^** | |
| No | 0 |
| Yes | 5 |
| **Renal replacement therapy ^c^** | |
| No | 0 |
| Yes | 4 |
|  | |
| **Total score** | 0-42 ^d^ |

Reproduced from a previous paper [7].

^a^ Continuous use of any vasopressor or inotrope.
^b^ Use of respiratory support, including invasive or non-invasive respiratory support and continuous use of continuous positive airway pressure (CPAP). Intermittent use of CPAP is not considered respiratory support. The PLOT-ICU study collected data on the use of mechanical ventilation only and did not consider non-invasive respiratory support and continuous CPAP.
^c^ Use of renal replacement therapy includes any renal replacement therapy whether chronic or acute, including continuous renal replacement therapy and intermittent haemodialysis, including the days in between intermittent haemodialysis.
^d^ Points assigned for the different variables in the score. It is not possible to obtain a total score of 1, 2 or 40 points. The worst value recorded during the first ICU day was used [7, 8].

# **Modified WHO bleeding classification**

| **Modified World Health Organization bleeding classification** |
| --- |
| **Grade 1: Minor Blood Loss**   - Petechiae (<2 mm in size) - Purpura (< 2.5 cm diameter/1 inch) - Subconjunctival bleeding - Upper airways: Oropharyngeal bleeding, epistaxis <30 minutes, not requiring packing - Abnormal vaginal bleeding (non-menstrual; < 2 pads/day) |
| **Grade 2: Mild Blood Loss**   - Retinal bleeding without visual impairment - Profuse epistaxis or oropharyngeal bleeding >30 minutes or requiring packing - Haemoptysis, blood in broncho-pulmonary lavage, blood in the tracheal tube (intubated patients) - Haematemesis, blood in nasogastric aspirates, melaena or haematochezia - Haematuria - Abnormal vaginal bleeding (>2 pads/day) - Diffuse petechia/purpura, multiple haematomas or ecchymoses, each >2.5 cm or any one >10 cm - Musculoskeletal bleeding, soft tissue bleeding and bleeding in cavity fluids evident macroscopically - Abnormal bleeding from invasive- or procedure sites |
| **Grade 3: Severe Blood Loss**   - CNS bleeding visible on imaging study but without neurological symptoms or clinical consequences - Any bleeding requiring up to two RBC transfusions within 24 hours of onset including epistaxis, oropharyngeal bleeding, haemoptysis, melaena, haematemesis, haematuria, haematochezia, vaginal bleeding, musculoskeletal bleeding, soft tissue bleeding and bleeding from invasive- or procedure sites |
| **Grade 4: Debilitating Blood Loss**   - Debilitating or life-threatening bleeding; including any bleeding requiring either more than two units of RBC, intubation and mechanical ventilation or surgical intervention (including coiling) within 24 hours after onset. - Non-fatal CNS bleeding resulting in neurological symptoms - Retinal bleeding with visual impairment - Fatal bleeding from any source |

Adapted from previous papers [3, 4, 9].

The WHO bleeding classification has been used in modified versions to grade bleeding events in large multicentre trials assessing platelet transfusions in patients with haematological malignancy and hypo-proliferative thrombocytopenia [10–13]. We used specific descriptors for each grade as previously used in these trials and adopted the classification to the intensive care unit setting [3, 4, 9].

# **Outcomes and reporting**

**Primary outcome**

1. Number of patients receiving at least one platelet transfusion in ICU categorized according to the type of product received (i.e., number of patients receiving pooled whole-blood-derived platelet transfusion, number of patients receiving apheresis derived platelet transfusion, and number of patients receiving both types). The outcome was reported overall and stratified on countries with ≥10 patients receiving platelet transfusion in ICU.

**Secondary platelet transfusion outcomes**

1. Number of pooled whole-blood-derived-platelet transfusions and apheresis derived platelet transfusions administered. The outcome was reported overall and stratified on countries with ≥10 transfusions and on sites with at least ≥10 transfusions within countries with ≥2 sites with ≥ 10 transfusions.
2. Volume (mL) contained per platelet transfusion. The outcome was reported overall and stratified on countries with ≥10 transfusions and on sites with ≥10 transfusions within countries with ≥2 sites with ≥ 10 transfusions.
3. Absolute post-transfusion platelet increments for prophylactic platelet transfusion administered in ICU as single transfusions (i.e., no other platelet transfusions took place on the day of the single prophylactic platelet transfusion). The outcome was reported overall and stratified on countries with ≥ 10 transfusions.
4. Number of sites using a fixed vs. weight-based approach to platelet dosing. Fixed dosing was defined as a “one size fits all” approach in which the administered platelet dose was independent of bodyweight. Weight based dosing was defined as dosing adjusted for the patient’s bodyweight.
5. Timing of platelet transfusions relative to the time from ICU admission. This outcome was reported graphically using heatmaps showing the timing and quantity of platelet transfusions relative to the time from ICU admission. It was reported for all platelet transfusions used in ICU and stratified on indications (prophylactic, pre-procedural and therapeutic).

**Secondary clinical outcomes**All clinical outcomes were stratified according to platelet transfusion status in ICU and severe thrombocytopenia (nadir platelet count <50x10^9^/L)

1. 90-day mortality
2. Number of patients with at least one major bleeding in ICU defined as WHO-grade 3 or 4 bleeding (see above).
3. Number of patients with at least one new thrombosis in the ICU including any arterial or venous thromboembolism; peripheral arterial thrombosis, acute coronary thrombosis, acute mesenteric ischemia, acute ischaemic stroke, central vein thrombosis, pulmonary embolus, peripheral venous thrombosis, and cerebral vein thrombosis
4. Number of patients receiving at least one RBC transfusion during ICU admission; either in ICU or operating rooms during ICU admission
5. Number of patients receiving at least one plasma transfusion; either in the ICU or in operating rooms during ICU admission. Includes any type of plasma product (fresh frozen plasma, cryoprecipitate, cryo-depleted plasma and octaplasLG®)

# **Missing data**

**Patients transferred to non-participating ICUs**

Overall, 13/504 (2.6%) patients were transferred to sites not participating in the PLOT-ICU study during the 90-day study period, where data on daily variables could not be obtained. We analysed these patients under the assumptions that no events had occurred during these periods as done previously [3].

**Platelet transfusion volumes**

Platelet transfusions volumes were missing for 15/618 (2.4%) transfusions; 10/565 (1.8%) platelet transfusions used in ICU and 5/53 (9.4%) transfusions used in operating rooms. All missing platelet transfusion volumes occurred in the same patient.

**Platelet count increments**

Platelet count increments were not possible to calculate on 22/183 ICU days in which a single prophylactic platelet transfusion was administered (i.e., no other platelet transfusions were administered during these days). In all these cases, the platelet count on the day *after* transfusion was not available for computations. In 18/22 (81.8%) of the cases, this was due to the transfusions being administered on the last day in ICU (i.e., either due to the patient dying or being discharged from ICU on the day of transfusion), in 2/22 (9.1%) of the cases, the patient died the day after transfusion without any biochemistry available before death, and in 2/22 (9.1%) of the cases, the were no platelet count available on the day after transfusion despite the patient being alive and still admitted to ICU.

# **Platelet transfusion used in operating rooms**

In total, 26 patients received a total of 53 platelet transfusions in operating rooms during their ICU stay; 9 patients receiving a total of 18 transfusions in operating rooms did not receive platelet transfusion in ICU and 17 patients receiving a total of 35 platelet transfusions in operating rooms also received platelet transfusion in ICU. Of these 17 patients, 14 patients had severe thrombocytopenia (<50x10^9^/L) while 3 patients had non-severe thrombocytopenia. Transfusions used in operating rooms were quantified by number and volume, but data regarding product type and indication for transfusion was not collected in the PLOT-ICU study [3].

# **eTable 1: Primary outcome stratified on country**

|  | **DEN**  **(n = 20)** | **FRA**  **(n = 25)** | **GER**  **(n = 12)** | **ESP**  **(n = 11)** | **GBR**  **(n = 12)** | **USA**  **(n = 14)** |
| --- | --- | --- | --- | --- | --- | --- |
| Pooled products | 18  (90.0%) | 8  (32.0%) | 9  (75.0%) | 11  (100.0%) | 8  (66.7%) | 1  (7.1%) |
| Apheresis product | 0  (0.0%) | 5  (20.0%) | 3  (25.0%) | 0  (0.0%) | 2  (16.7%) | 13  (92.9%) |
| Both products | 2  (10.0%) | 12 (48.0%) | 0  (0.0%) | 0  (0.0%) | 2  (16.7%) | 0  (0.0%) |

Number (percentage) of patients receiving pooled, apheresis and both product types stratified on countries with ≥10 patients receiving platelet transfusion in ICU; 1 patient in Finland received pooled products, 5 patients in Norway received pooled products and 2 received both products, 1 patient in Portugal received pooled products, and 2 patients in Sweden received pooled products.

Platelet transfusions used in operating rooms were not accounted for as data on product type were not available; in total 17/105 (16.2%) patients who received platelet transfusions in ICU also received 35 platelet transfusions in operating rooms; DEN: 5 (25.0%), FRA: 2 (8.0%), GER: 1 (8.3%), ESP: 1 (9,1%), GBR: 3 (25%), USA: 1 (7.1%). In addition, 9 patients who did not receive platelet transfusion in ICU received 18 platelet transfusions in operating rooms.

Abbreviations: DEN (Denmark), FRA (France), GER (Germany), ESP (Spain), GBR (United Kingdom), USA (United States of America)

# **eFigure 1. Type of platelet transfusion used in ICU across countries**


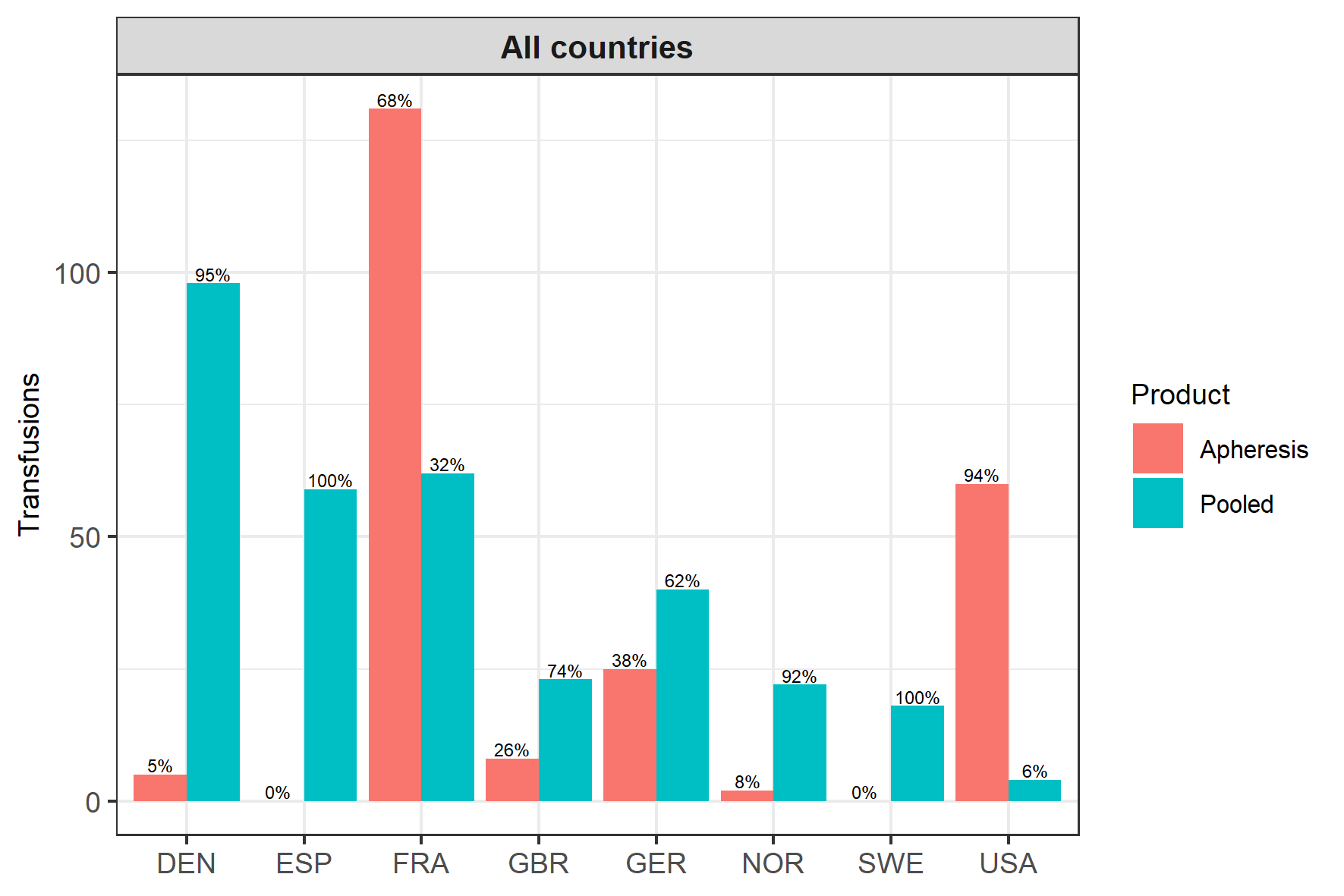


Number of pooled and apheresis products used in ICU stratified on countries. Percentages above the bars represent proportions within countries. Only countries with ≥10 platelet transfusions are shown; we recorded 7 and 1 pooled products in Finland and Portugal, respectively (not shown).

Abbreviations: DEN (Denmark), ESP (Spain), FRA (France), GBR (United Kingdom), GER (Germany), NOR (Norway), SWE (Sweden), USA (United States of America)

# **eFigure 2. Type of platelet transfusion used in ICU across sites within countries**


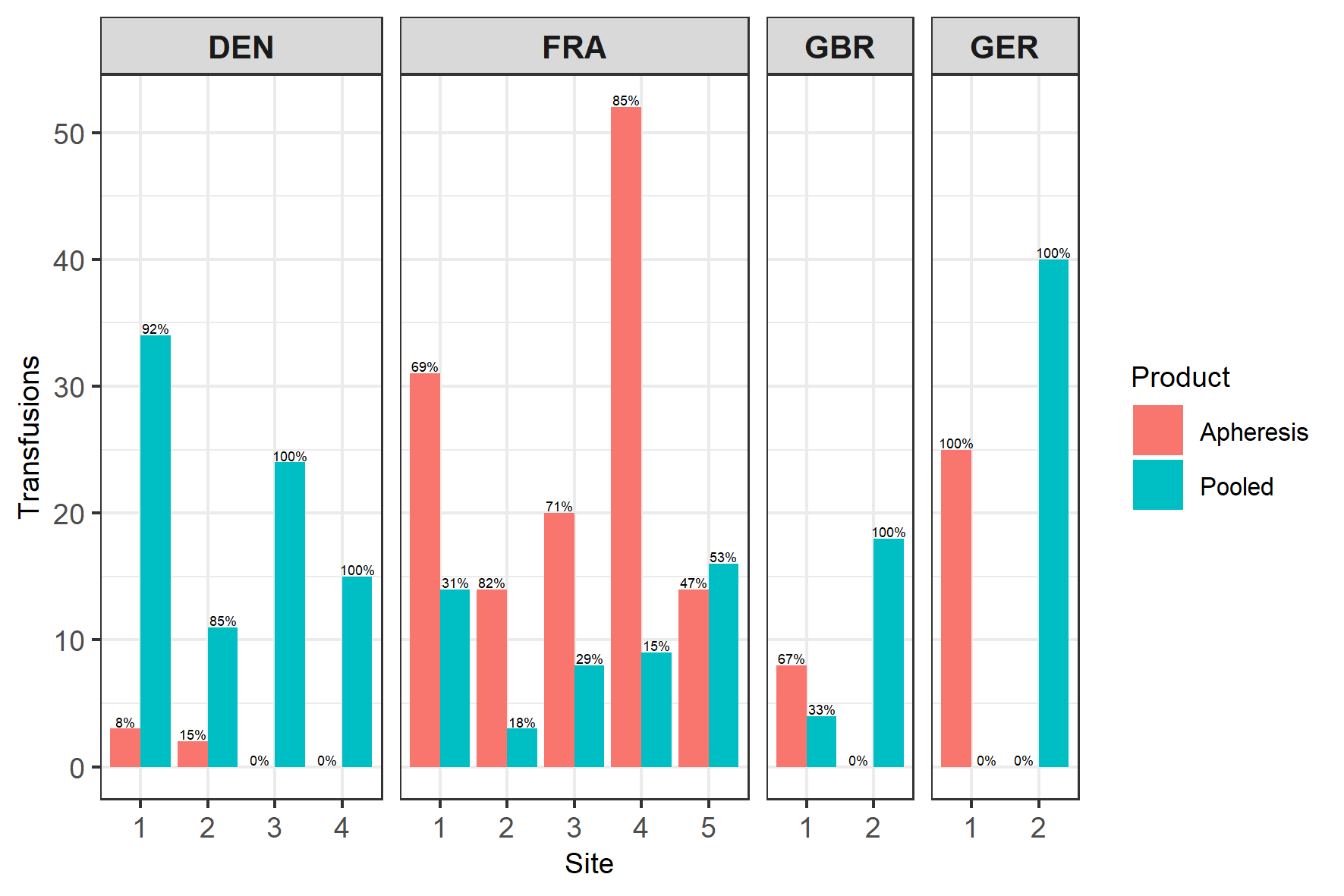


Number of pooled and apheresis products used in ICU stratified on country and site. Percentages above the bars represent proportions within sites. Only sites with ≥10 transfusions in countries with at least 2 sites with ≥10 transfusions are shown; 4 additional Danish sites administered 14 pooled products, 3 Spanish sites administered 59 pooled products, 1 Finish site administered 7 pooled products, 4 additional French sites administered 12 pooled products, 1 additional site in United Kingdom administered 1 pooled product, 3 Norwegian sites administered 22 pooled products and 2 apheresis products, 1 Portuguese site administered 1 pooled product and, 1 Swedish site administered 18 pooled products, and 2 sites in United States used 4 pooled products and 60 apheresis products.

Abbreviations: DEN (Denmark), FRA (France), GBR (United Kingdom), GER (Germany)

# **eFigure 3. Relative distributions of volumes for platelet transfusions used in ICU across countries**


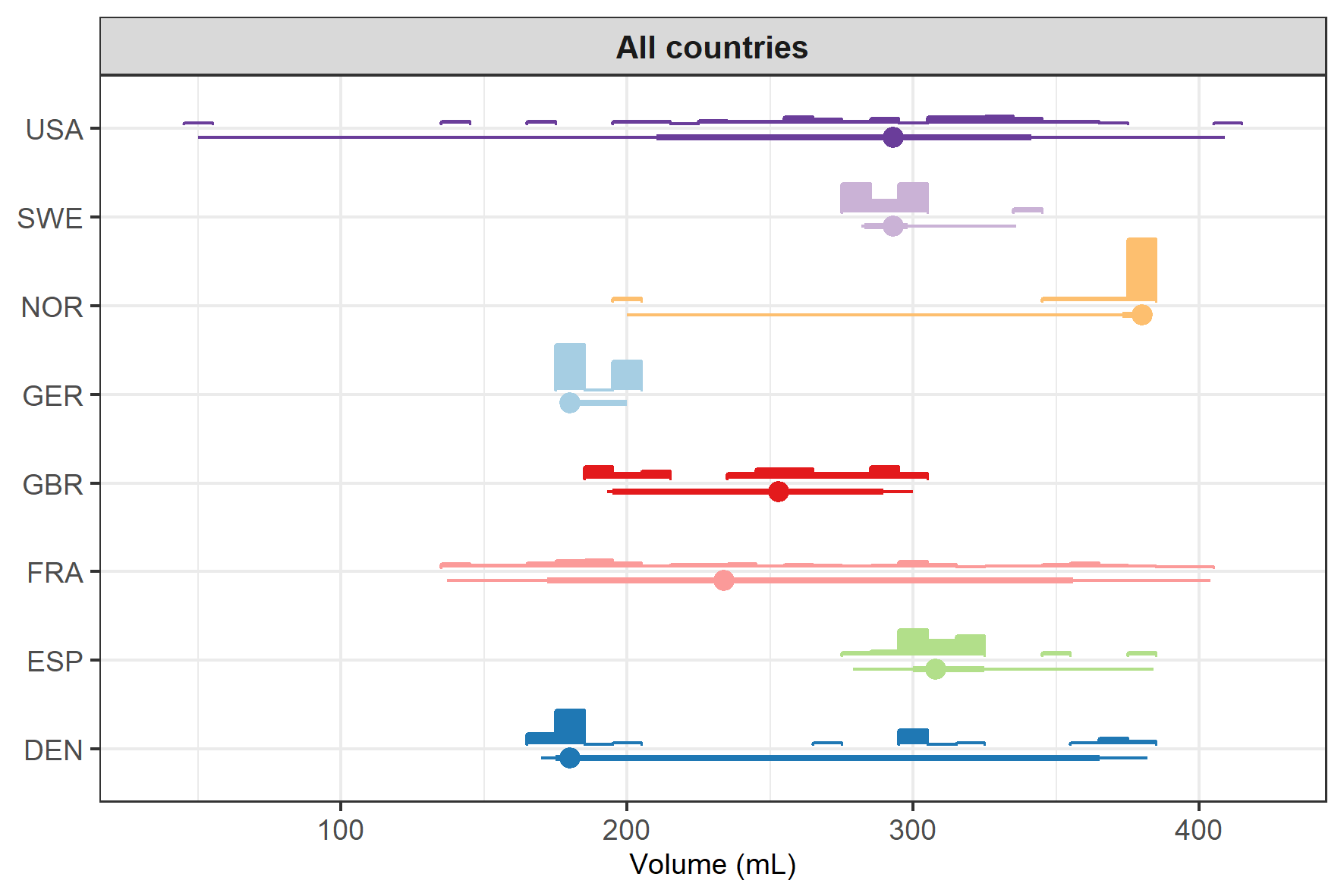


Relative distributions of volumes for 555 platelet transfusions in ICU stratified on country. Below each distribution is a horizontal box plot: thin lines represent ranges, thick lines represent IQRs, and dots represent medians. Volume data were missing for 10/565 (1.8%). Only countries with ≥10 transfusions are shown; 1 transfusion with a volume of 300 mL and 7 transfusions, all with a volume of 250 mL, were registered in Portugal and Finland, respectively (data not shown).

Abbreviations: DEN (Denmark), ESP (Spain), FRA (France), GBR (United Kingdom), GER (Germany), NOR (Norway), SWE (Sweden), USA (United States of America)

# **eFigure 4. Relative distributions of volumes for platelet transfusions used in ICU across sites**


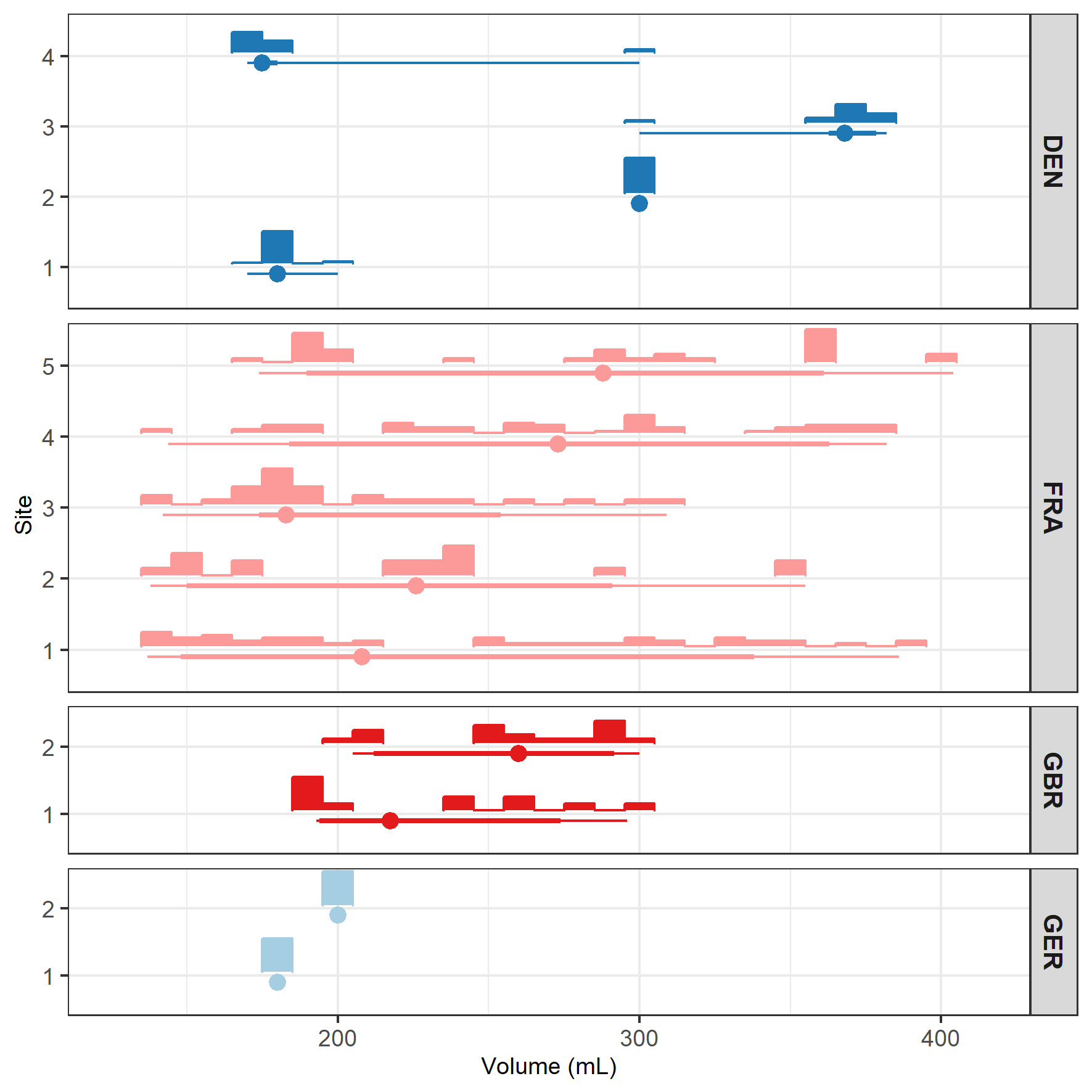


Relative distributions of volumes for 365 platelet transfusions in ICU stratified on site. Below each distribution is a horizontal box plot: thin lines represent ranges, thick lines represent IQRs, and dots represent medians. No volume data were missing for these sites. Only sites ≥10 transfusions in countries with at least 2 sites with ≥10 transfusions are shown. Median (IQR) volumes for sites not shown were: 4 additional Danish sites (n=14 transfusions); 287 (180 to 300) mL, 3 Spanish sites (n=59, missing data for 10/59); 308 (300 to 325) mL, 1 Finish site (n=7); 250 (250 to 250) mL, 4 additional French sites (n=12); 200 (200 to 207) mL, 3 Norwegian sites (n=24); 380 (380 to 380), 1 Swedish site (n=18), 293 (283 to 297) mL, 2 sites in United States (n=64); 293 (256 to 329) and 1 Portuguese site and 1 additional site in United Kingdom each administered 1 transfusion with a volume of 300 and 269 mL, respectively.

Abbreviations: DEN (Denmark), FRA (France), GBR (United Kingdom), GER (Germany)

# **eFigure 5. Relative distributions of volumes for platelet transfusions used in ICU and operating rooms across countries**


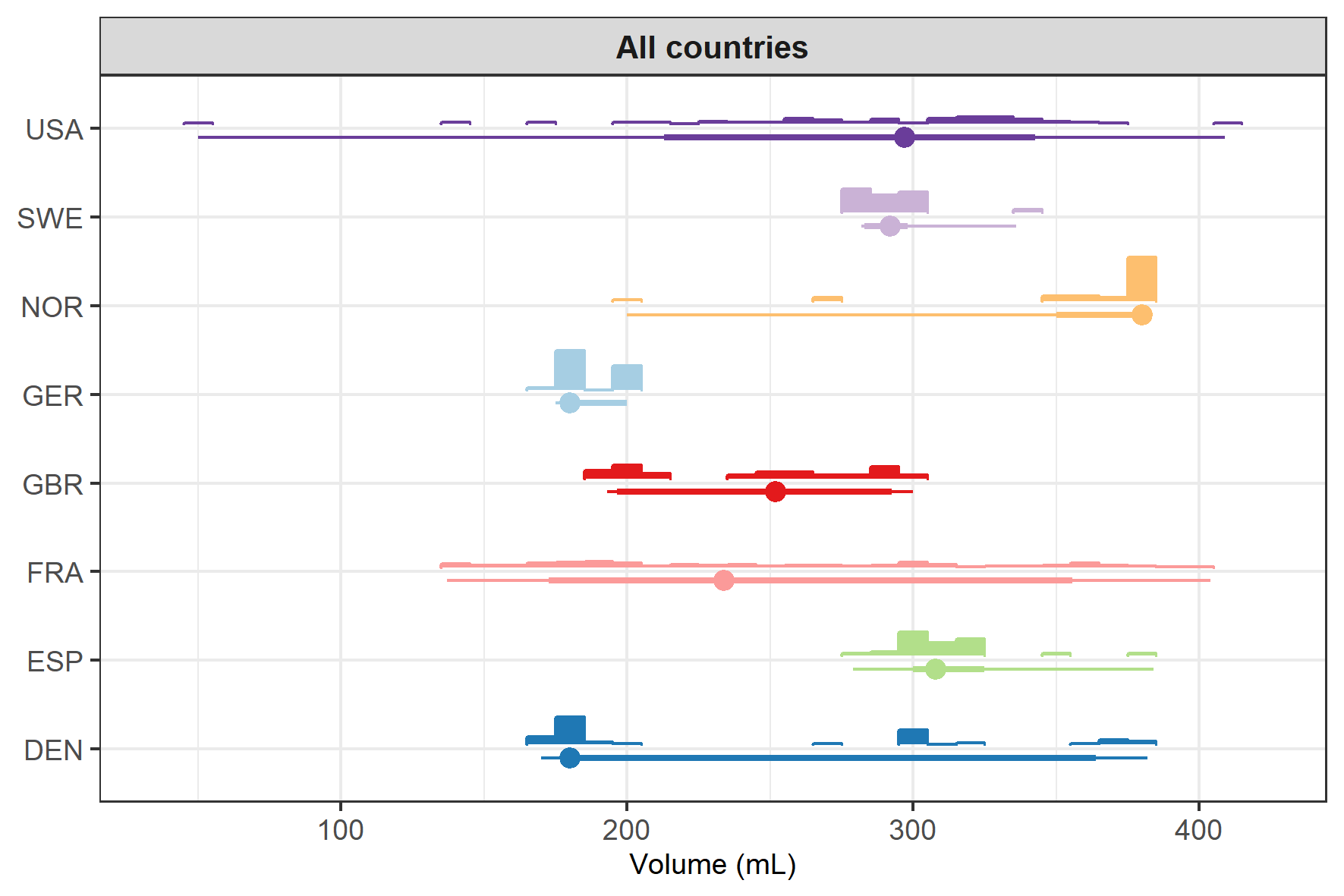


Relative distributions of 603 platelet transfusion volumes, including volumes for 48 platelet transfusions used in operating rooms, stratified by country. Below each distribution is a horizontal box plot: thin lines represent ranges, thick lines represent IQRs, and dots represent medians. Volume data were missing for 15/618 (2.4%) transfusions. Only countries ≥10 transfusions are shown; 1 transfusion with a volume of 300 mL and 7 transfusions, all with a volume of 250 mL were registered in Portugal and Finland, respectively (not shown). The overall median (IQR) was 260 (180 to 308) mL per transfusion.

Abbreviations: DEN (Denmark), ESP (Spain), FRA (France), GBR (United Kingdom), GER (Germany), NOR (Norway), SWE (Sweden), USA (United States of America)

# **eFigure 6. Relative distributions of volumes for platelet transfusions used in ICU and operating rooms across sites**


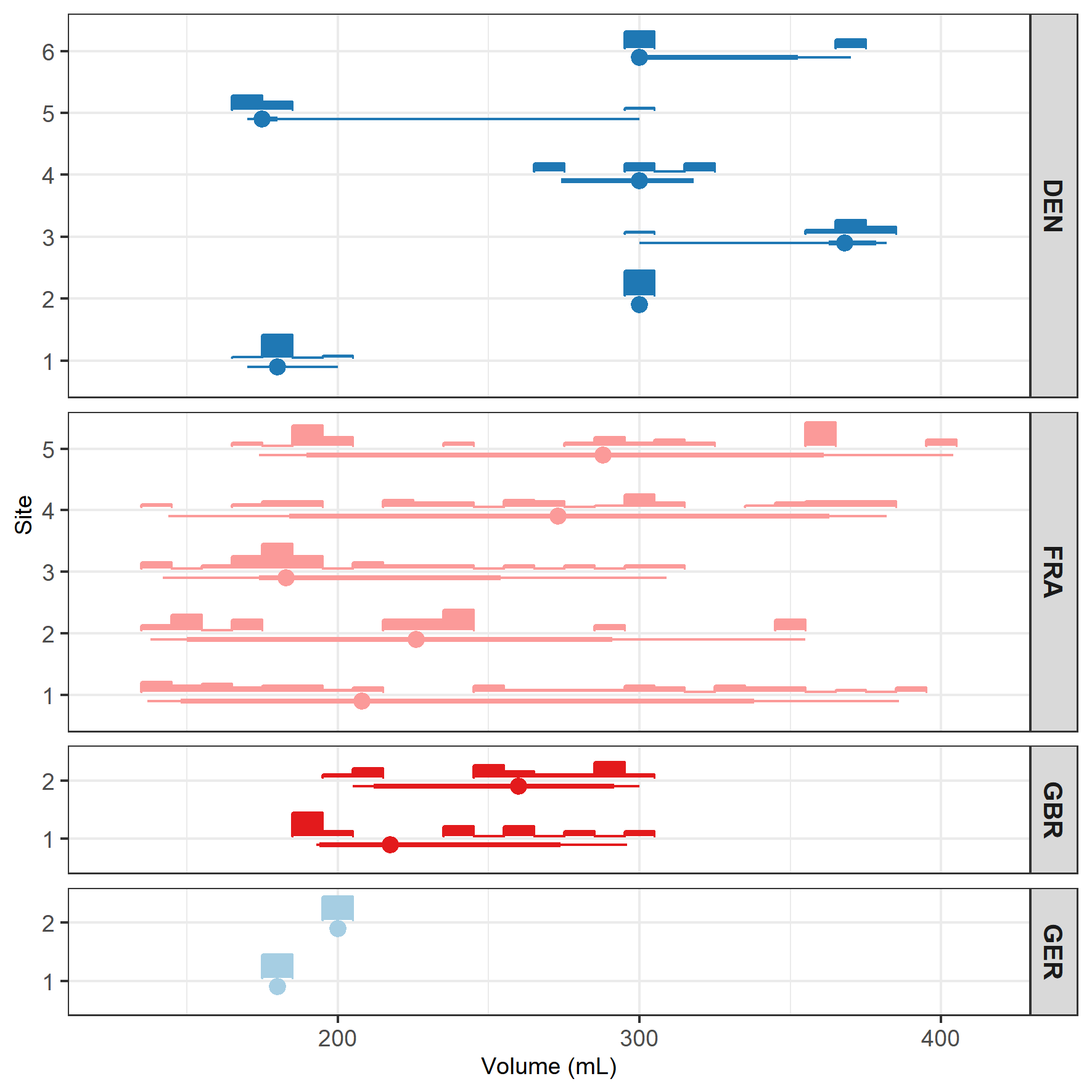


Relative distributions of 401 platelet transfusion volumes, including 27 platelet transfusion used in operating rooms, stratified on site. Only sites ≥10 transfusions in countries with at least two sites ≥10 transfusions are shown. Below each distribution is a horizontal box plot: thin lines represent ranges, thick lines represent IQRs, and dots represent medians. No volume data were missing for these sites. Median (IQR) volumes for sites not shown were: 3 additional Danish sites (n=7 transfusions); 180 (180 to 180) mL, 3 Spanish sites (n=65, missing data for 15/65); 308 (300 to 325) mL, 1 Finish site (n=7); 250 (250 to 250) mL, 4 additional French sites (n=12); 200 (200 to 207) mL, 4 Norwegian sites (n=34); 380 (364 to 380), 1 Swedish site (n=22), 292 (283 to 297) mL, 2 sites in United States (n=66); 297 (260 to 331), 1 Portuguese site administering 1 transfusion of 300 mL and 1 site in United Kingdom administered 3 transfusions of 200 mL, 200 mL and 234 mL, respectively.

Abbreviations: DEN (Denmark), FRA (France), GBR (United Kingdom), GER (Germany)

# **eFigure 7. Platelet count increments across countries**


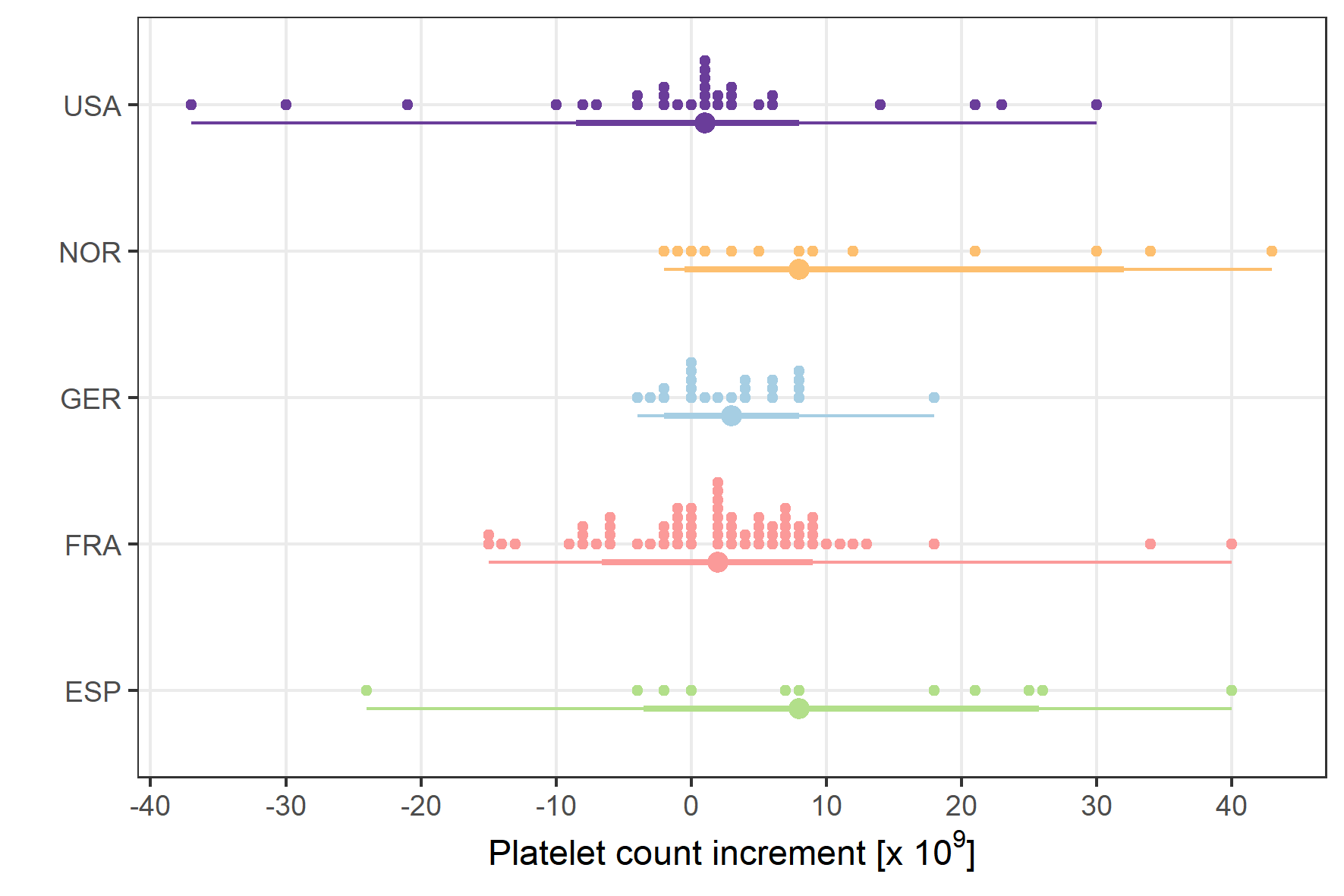


Platelet count increments for 146 prophylactic transfusions administered as single transfusions stratified by country. Small dots represent a platelet increment from a single transfusion. Below each distribution is a horizontal box plot: thin lines represent ranges, thick lines represent IQRs, and big dots represent medians. Only countries with at least 10 prophylactic transfusions administered as single transfusions with available data are shown; 7 single transfusions were observed in Denmark with a median (IQR) increment of 6 (4 to 13) x 10^9^/L, 6 in United Kingdom with an increment of 1 (0 to 6) x 10^9^/L and 2 in Sweden with increments of -9 and 17 respectively.

Abbreviations: ESP (Spain), FRA (France), GER (Germany), NOR (Norway), USA (United States of America)

# **eFigure 8. Number of patients receiving platelet transfusion in ICU or operating rooms according to days from ICU admission**


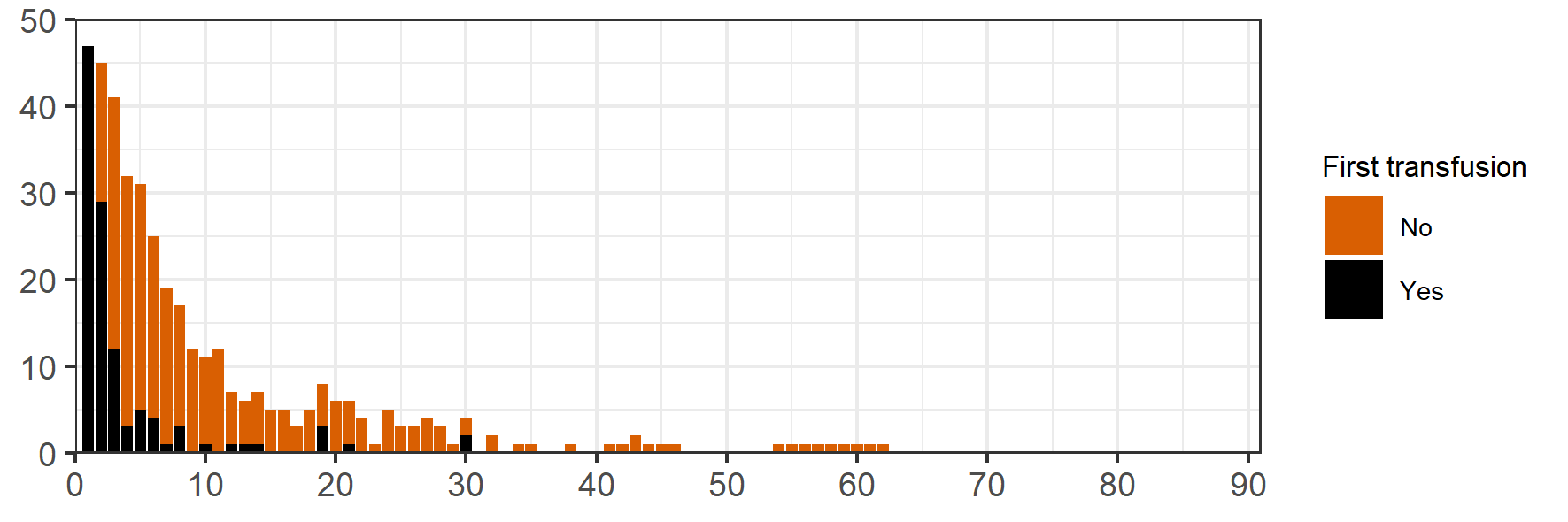


Day 1 represent the day of ICU admission. The black part of each bar represents patients who received their first platelet transfusion(s) on this day, the orange part represents patients who received a platelet transfusion on this day but previously had received another. The median number of days to first platelet transfusions were 2 (IQR 1 to 3). Among the 114 patients transfused with platelet transfusions (in ICU or operating rooms), 84.2% and 92.1% received their first platelet transfusion(s) within the first 5 and 10 days in ICU, respectively.

# **eFigure 9. Timing and number of prophylactic platelet transfusions in ICU**


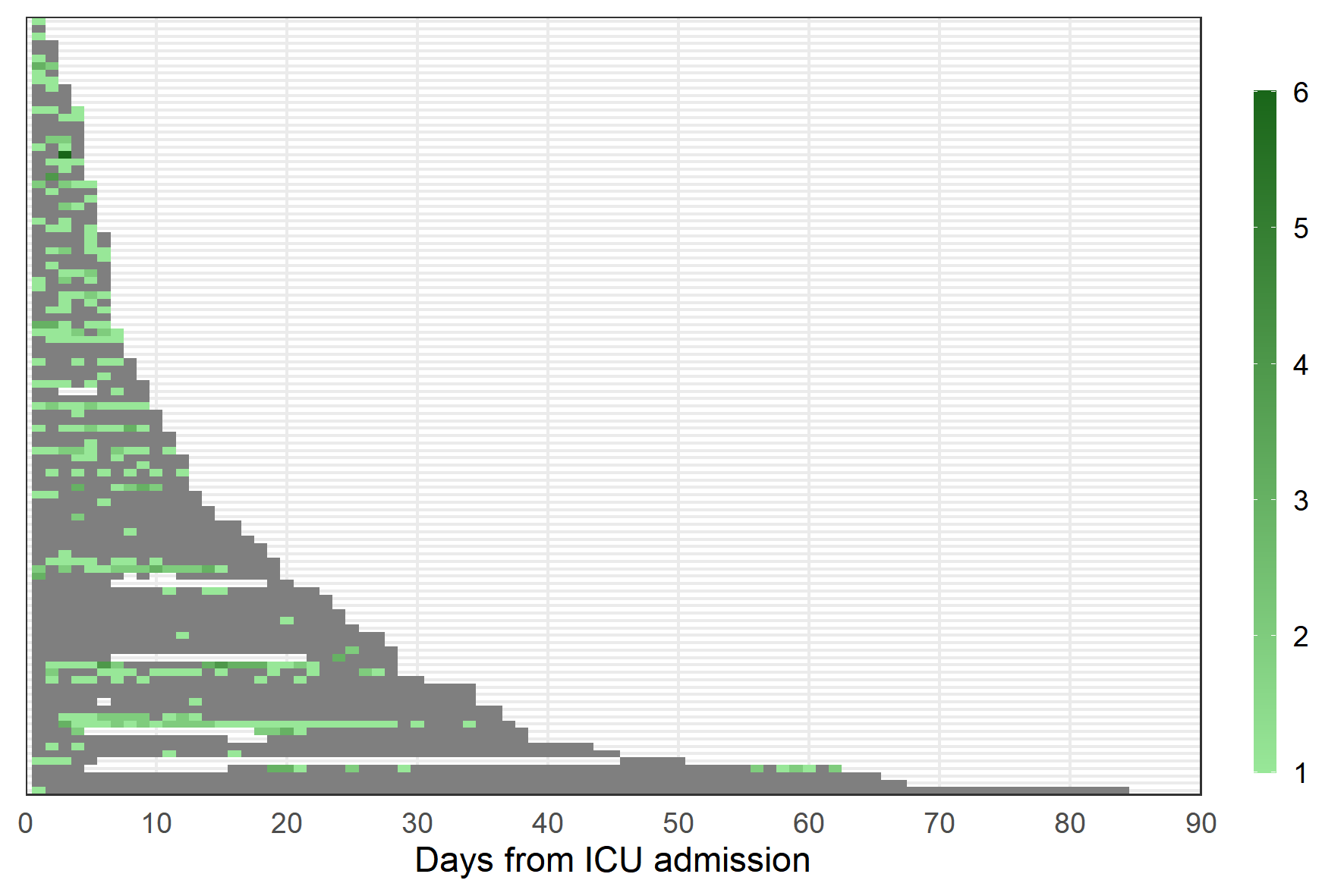


Timing and number of prophylactic platelet transfusions used among the 105 patients who received platelet transfusions in ICU. Each horizontal line of tiles represents a patient, the grey-coloured tiles represent days in the ICU where no platelets were transfused, and the green-coloured tiles represent days in the ICU where the patient was transfused with platelets to reduce the risk of bleeding. The colour intensity corresponds to the number of prophylactic platelet transfusions received that day. Among the 73 patients who received prophylactic platelet transfusion, the median number of days with prophylactic platelet transfusion was 2 (IQR 1 to 4) per patient and the number of prophylactic platelet transfusions administered on these days was 1 (1 to 2).

# **eFigure 10. Timing and number of therapeutic platelet transfusions in ICU**


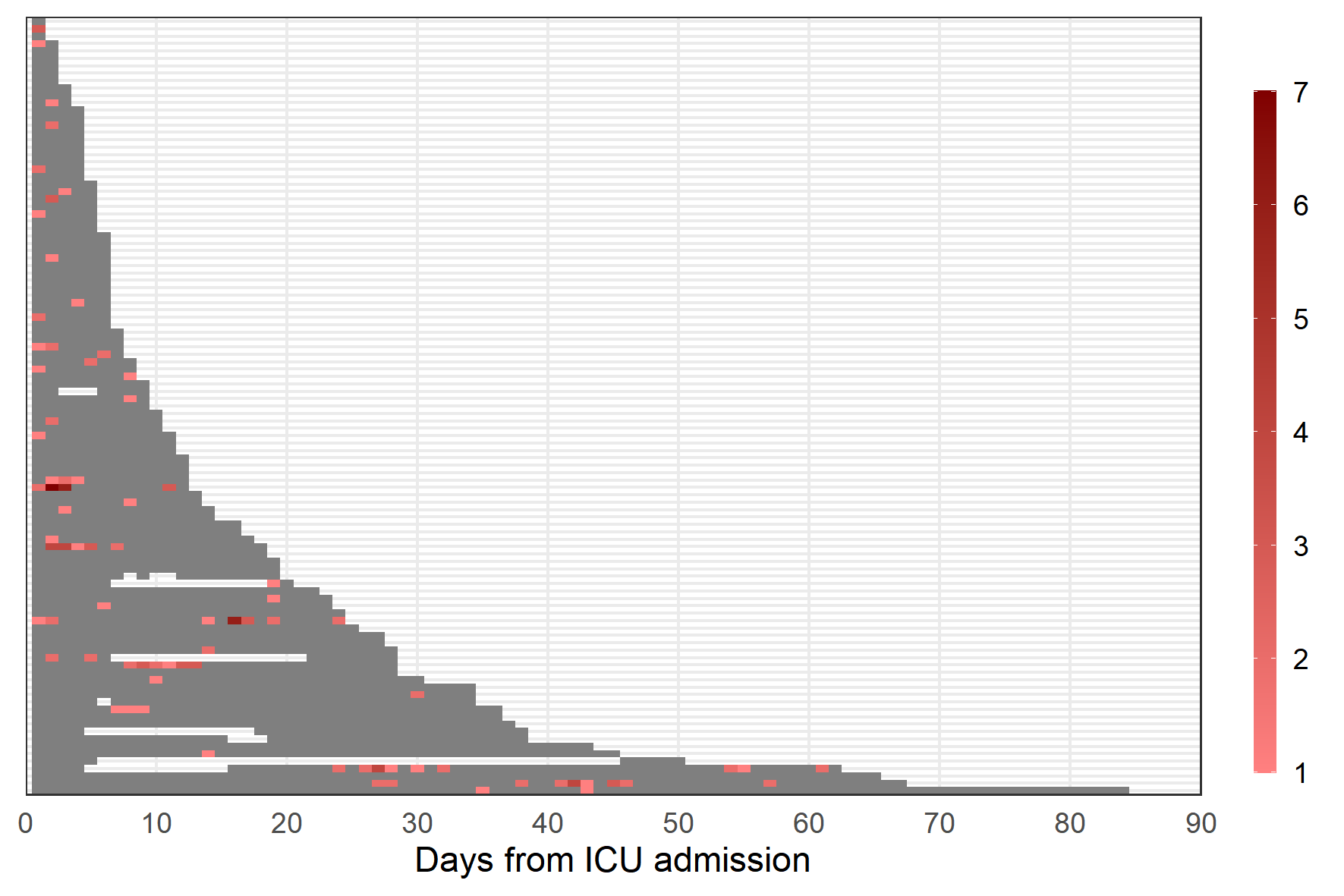


Timing and number of therapeutic platelet transfusions used among the 105 patients who received platelet transfusions in ICU. Each horizontal line of tiles represents a patient, the grey-coloured tiles represent days in ICU where no platelets were transfused, and the red-coloured tiles represent days in ICU where the patient was transfused with platelets to treat bleeding. The colour intensity corresponds to the number of therapeutic platelet transfusions received that day. Among the 39 patients who received therapeutic platelet transfusions, the median number of days with therapeutic platelet transfusion was 1 (IQR 1 to 2) per patient and the number of therapeutic platelet transfusions administered on these days was 2 (1 to 2).

# **eFigure 11. Timing and number of pre-procedural platelet transfusions in ICU.**


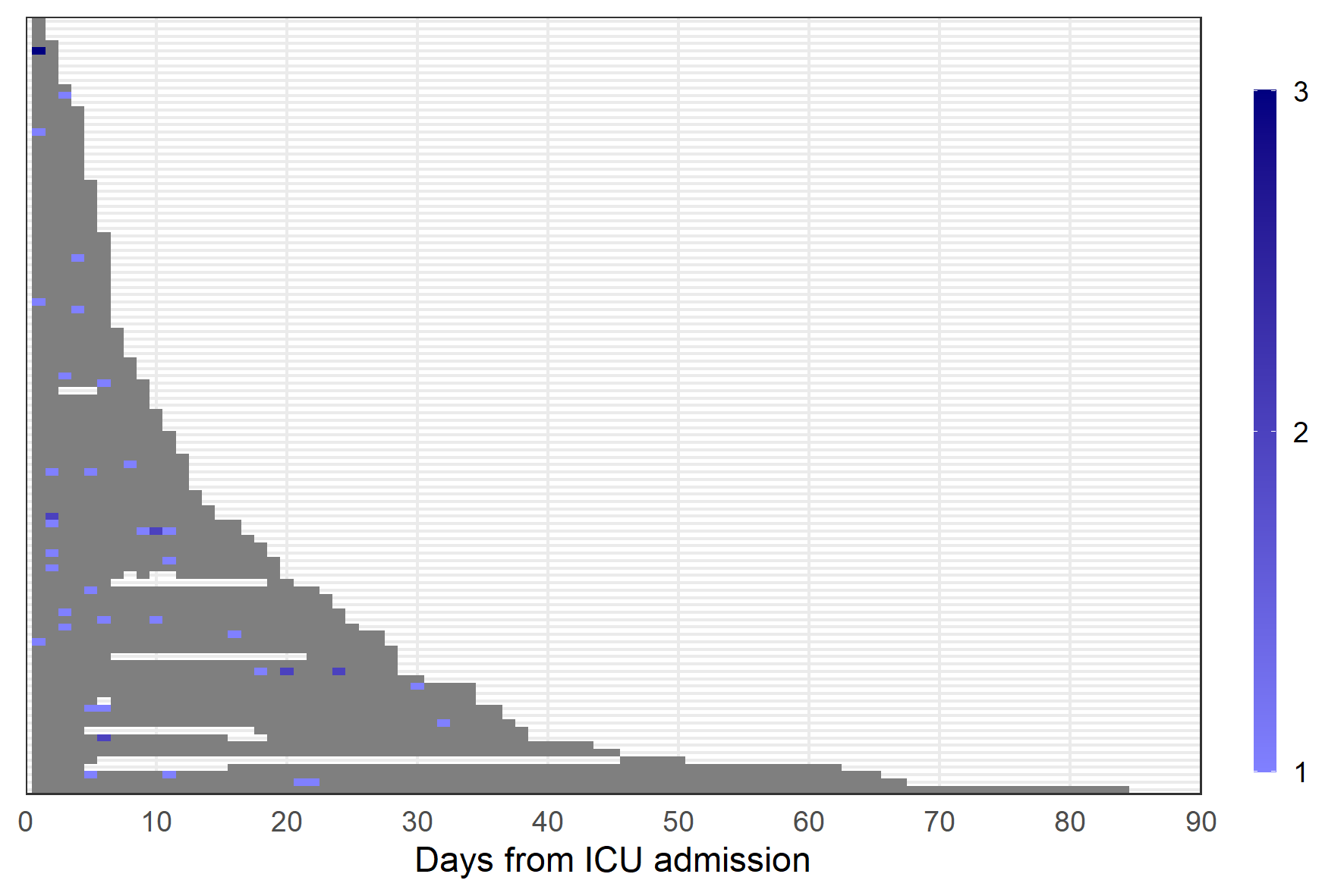


Timing and number of pre-procedural platelet transfusions used among the 105 patients who received platelet transfusions in ICU. Each horizontal line of tiles represents a patient, the grey-coloured tiles represent days in ICU where no platelets were transfused, and the blue-coloured tiles represent days in ICU where the patient was transfused with platelets to cover invasive procedures. The colour intensity corresponds to the number of pre-procedural platelet transfusions received that day. Among the 29 patients who received pre-procedural platelet transfusions, the median number of days with pre-procedural platelet transfusion was 1 (IQR 1 to 1) and the number of pre-procedural platelet transfusions administered on these days was 1 (1 to 1).

# **eFigure 12. Timing and number of platelet transfusions in ICU or operating rooms**


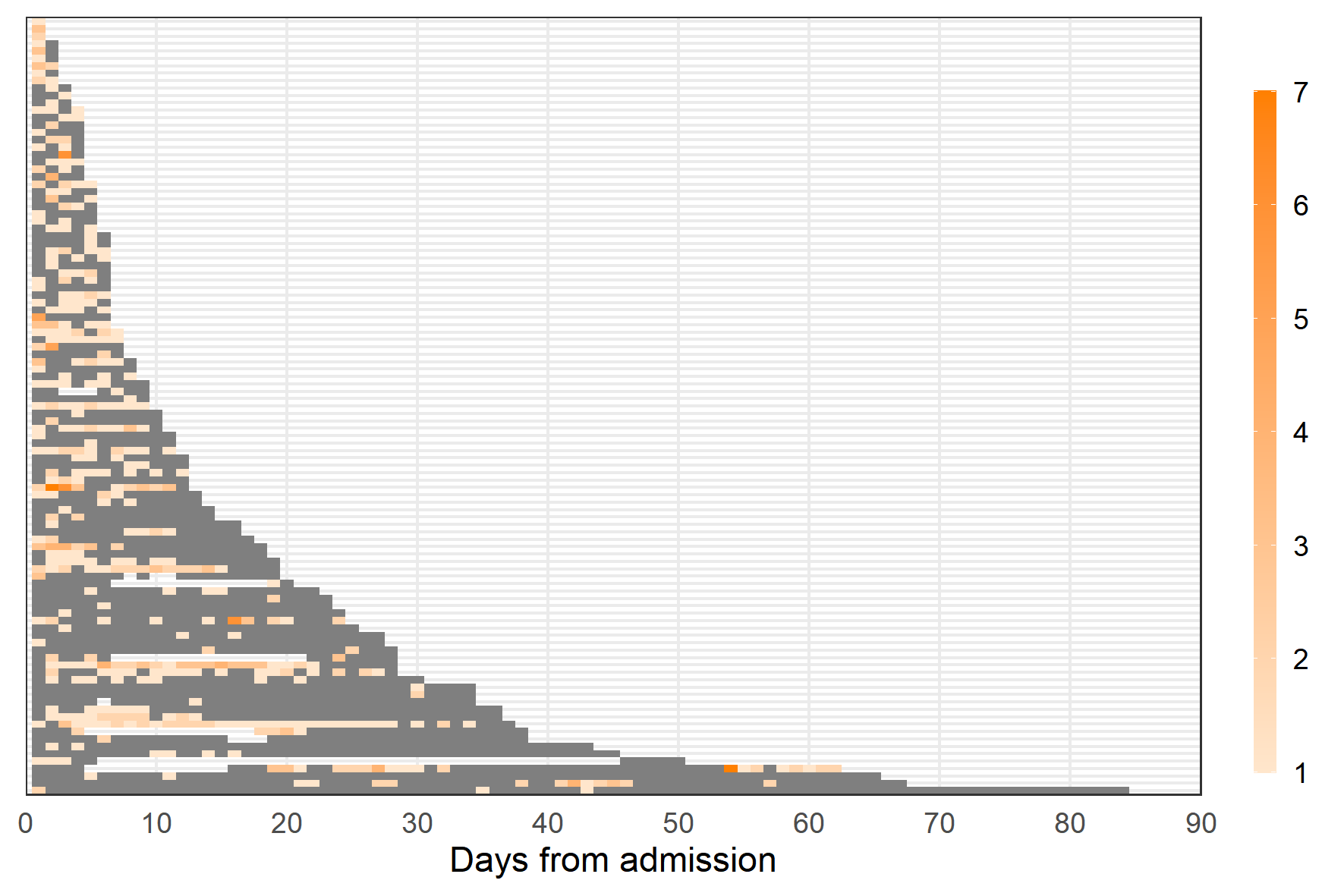


Timing and number of platelet transfusions used among the 114 patients who received platelet transfusions in ICU or operating room. Each horizontal line of tiles represents a patient, the grey-coloured tiles represent days in ICU where no platelets were transfused, and the orange-coloured tiles represent days in ICU where the patient was transfused with platelets. The colour intensity corresponds to the number of platelet transfusions received that day. The median number of days with platelet transfusion was 2 (IQR 1 to 4) per patient and the number of platelet transfusions administered on these days was 1 (1 to 2).

# **Additional survey results**

The survey was distributed by e-mail on the November 17^th^, 2023 and closed on January the 24^th^, 2024. Reminders were sent to non-responders every two weeks.

**Response rate**

A total of 42/43 (97.5%) centres responded to the survey.

**Product availability**

Among responders, apheresis products were available in 41 (97.6%) centres, pooled products in 39 (92.9%), and both products in 38 (90.5%).

**Preparation of pooled products**

Of the 39 centres using pooled products, the buffy coat method was employed in 32 (82.1%) centres, the platelet rich plasma method in 6 (15.4%) and the single centrifugation method in 1 (2.6%) [14]. The platelet rich plasma method was used in 4 (10.3%) centres in France, 1 (2.6%) in Norway and 1 (2.6%) in Spain. A single Norwegian centre (2.6%) employed the single centrifugation method.

**Platelet dosing**

Among responders, 31 (73.8%) centres employed fixed dosing, while 11 (26.2%) employed weight-based dosing. All 10 (23.8%) centres in France and a single centre in Spain (2.4%) employed weight-based dosing. All reported dosing practices is presented in the (eTable 2).

# **eTable 2: Overview of platelet dosing**

| **Site** | **Method of dosing** | **Median or target dose platelet dose** | |
| --- | --- | --- | --- |
|  |  | **Pooled products** | **Apheresis products** |
| GER01 | Fixed | Not used. | 2.41^e^ |
| GER02 | Fixed | 2.00 | 2.00 |
| DEN01 | Fixed | 1.98 | 2.15 |
| DEN02 | Fixed | 2.73 | 2.39 |
| DEN03 | Fixed | 2.73 | 2.39 |
| DEN07 | Fixed | 1.98 | 2.15 |
| DEN08 | Fixed | 1.98 | 2.15 |
| DEN09 | Fixed | 1.98 | 2.15 |
| DEN10 | Fixed | 2.51 | 2.34 |
| DEN11 | Fixed | 2.72 | 2.77 |
| DEN12 | Fixed | 2.67 | 2.47 |
| DEN13 | Fixed | 1.98 | 2.15 |
| FIN01 | Fixed | 2.69^e^ | 2.39^e^ |
| FRA01 | Weight-based | Target: 0.5 to 0.7 per 10 kg actual body weight | |
| FRA02 | Weight-based | Target: 0.5 to 0.7 per 10 kg actual body weight | |
| FRA03 | Weight-based | Target: 0.5 to 0.7 per 10 kg actual body weight | |
| FRA04 | Weight-based | Target: 0.5 to 0.7 per 10 kg actual body weight | |
| FRA05 | Weight-based | Target: 0.7 per 10 kg actual body weight | |
| FRA06 | Weight-based | Target: 0.5 to 0.7 per 10 kg actual body weight | |
| FRA07 | Weight-based | Target: 0.5 per 10 kg actual body weight | |
| FRA08 | Weight-based | Target: 0.5 to 0.7 per 10 kg actual body weight | |
| FRA09 | Weight-based | Target: 0.5 per 10 kg actual body weight | |
| FRA10 | Weight-based | Target: 0.5 to 0.7 per 10 kg actual body weight | |
| NOR01^a^ | Fixed | NA | NA |
| NOR02^a^ | Fixed | NA | NA |
| NOR03 | Fixed | 2.77^e^ | 2.76^e^ |
| NOR04 | Fixed | 3.15^e^ | 2.94^e^ |
| NOR05 | Fixed | 3.00^e^ | 2.89^e^ |
| NOR06 | Fixed | 2.73^e^ | 2.78^e^ |
| POR01 | Fixed | 2.40^e^ | 2.00^e^ |
| POR02^b^ | NA | NA | NA |
| POR03 | Fixed | 2.75^e^ | 3.00^e^ |
| POR04 | Fixed | >2.50^f^ | >2.50^f^ |
| POR05 | Fixed | >2.50^f^ | >2.50^f^ |
| SWE01 | Fixed | 2.39 | 2.60 |
| ESP01 | Fixed | 3.00 | 3.51 |
| ESP02^c^ | Weight-based | 3.46 | Not used |
| ESP03 | Fixed | 3.50 | >2.50^f^ |
| GBR01 | Fixed | 3.12^e^ | 2.54^e^ |
| GBR02 | Fixed | 3.12^e^ | 2.54^e^ |
| GBR03 | Fixed | 2.84 | 2.74 |
| USA01^d^ | Fixed | Not used | 3.00 |
| USA02 | Fixed | Not used | 3.50 |

Sites are indicated by country abbreviation and number. Platelet doses are number of platelets contained in a single adult transfusion x10^11^. Among responders, platelet dose data for pooled and apheresis products suitable for aggregation could not be obtained for 4 (9.5%; NOR01, NOR02, POR04, POR05) and 5 sites (12%; NOR01, NOR02, POR04, POR05, ESP03), respectively.

^a^ Irrespective of product, platelet doses were defined according to a therapeutic dose of (range) 2.20 to 3.39x10^11^ platelets at this site. Patients often received 1.5 therapeutic dose containing (range) 3.40 to 4.39x10^11^ platelets. Mean/median doses were not available. These data were not suitable for aggregation.
^b^ Did not respond to the survey.
^c^ Patients received 1 pooled product containing a mean of 3.46x10^11^ platelets if the patient’s weight was ≤ 90 kg and 2 pooled products if the patient’s weight was > 90 kg.
^d^ Pooled products were available during the PLOT-ICU study but had since been phased out.
^e^ Reported as mean dose. We assumed that the underlying data were normally distributed and interpreted these as medians to facilitate data aggregation.
^f^ Reported as minimal accepted value in the respective products. These data were not suitable for aggregation.

Abbreviations: NA (missing), DEN (Denmark), FIN (Finland), FRA (France), NOR (Norway), POR (Portugal), SWE (Sweden), ESP (SPAIN), GBR (United Kingdom), USA (United States of America).

# **References**

1. Elm E von, Altman DG, Egger M, et al (2007) Strengthening the Reporting of Observational Studies in Epidemiology (STROBE) statement: guidelines for reporting observational studies. BMJ 335:806–808. https://doi.org/10.1136/BMJ.39335.541782.AD

2. Vandenbroucke JP, Von Elm E, Altman DG, et al (2007) Strengthening the Reporting of Observational Studies in Epidemiology (STROBE): explanation and elaboration. PLoS Med 4:1628–1654. https://doi.org/10.1371/JOURNAL.PMED.0040297

3. Anthon CT, Pène F, Perner A, et al (2023) Thrombocytopenia and platelet transfusions in ICU patients: an international inception cohort study (PLOT-ICU). Intensive Care Med 49:1327–1338. https://doi.org/10.1007/s00134-023-07225-2

4. Anthon CT, Pène F, Perner A, et al (2022) Platelet transfusions and thrombocytopenia in intensive care units: Protocol for an international inception cohort study (PLOT‐ICU). Acta Anaesthesiol Scand 66:1146–1155. https://doi.org/10.1111/aas.14124

5. Singer M, Deutschman CS, Seymour CW, et al (2016) The Third International Consensus Definitions for Sepsis and Septic Shock (Sepsis-3). JAMA 315:801. https://doi.org/10.1001/jama.2016.0287

6. Bernal W, Wendon J (2013) Acute liver failure. N Engl J Med 369:2525–34. https://doi.org/10.1016/j.bpg.2013.08.010

7. Granholm A, Perner A, Krag M, et al (2018) Development and internal validation of the Simplified Mortality Score for the Intensive Care Unit (SMS-ICU). Acta Anaesthesiol Scand 62:336–346. https://doi.org/10.1111/aas.13048

8. Granholm A, Perner A, Krag M, et al (2019) External validation of the Simplified Mortality Score for the Intensive Care Unit (SMS-ICU). Acta Anaesthesiol Scand 63:1216–1224. https://doi.org/10.1111/aas.13422

9. Anthon CT, Pène F, Perner A, et al (2023) Platelet transfusions in adult thrombocytopenic <scp>ICU</scp> patients: Protocol for a sub‐study of the <scp>PLOT‐ICU</scp> cohort. Acta Anaesthesiol Scand. https://doi.org/10.1111/aas.14365

10. Stanworth SJ, Estcourt LJ, Powter G, et al (2013) A no-prophylaxis platelet-transfusion strategy for hematologic cancers. N Engl J Med 368:1771–1780. https://doi.org/10.1056/NEJMoa1212772

11. Wandt H, Schaefer-Eckart K, Wendelin K, et al (2012) Therapeutic platelet transfusion versus Routine prophylactic transfusion in patients with haematological malignancies: An open-label, multicentre, randomised study. Lancet 380:1309–1316. https://doi.org/10.1016/S0140-6736(12)60689-8

12. Heddle NM, Cook RJ, Tinmouth A, et al (2009) A randomized controlled trial comparing standard- and low dose strategies for transfusion of platelets (SToP) to patients with thrombocytopenia. Blood 113:1564–1573. https://doi.org/10.1182/blood-2008-09-178236.The

13. Slichter SJ, Kaufman RM, Assmann SF, et al (2010) Dose of prophylactic platelet transfusions and prevention of hemorrhage. N Engl J Med 362:600–613. https://doi.org/10.1056/NEJMoa0904084

14. (2023) The Guide to the preparation, use and quality assurance of blood components, 21st ed. European Directorate for the Quality of Medicines & HealthCare of the Council of Europe (EDQM)
